# Nutritional Modulation of Host Defense Peptide Synthesis: A Novel Host-Directed Antimicrobial Therapeutic Strategy?

**DOI:** 10.1016/j.advnut.2024.100277

**Published:** 2024-07-23

**Authors:** Melanie Whitmore, Isabel Tobin, Amanda Burkardt, Guolong Zhang

**Affiliations:** Department of Animal and Food Sciences, Oklahoma State University, Stillwater, OK, United States

**Keywords:** host defense peptides, antimicrobial peptides, antimicrobial resistance, host-directed antimicrobial therapy, nutritional regulation

## Abstract

The escalating threat of antimicrobial resistance underscores the imperative for innovative therapeutic strategies. Host defense peptides (HDPs), integral components of innate immunity, exhibit profound antimicrobial and immunomodulatory properties. Various dietary compounds, such as short-chain fatty acids, vitamins, minerals, sugars, amino acids, phytochemicals, bile acids, probiotics, and prebiotics have been identified to enhance the synthesis of endogenous HDPs without provoking inflammatory response or compromising barrier integrity. Additionally, different classes of these compounds synergize in augmenting HDP synthesis and disease resistance. Moreover, dietary supplementation of several HDP-inducing compounds or their combinations have demonstrated robust protection in rodents, rabbits, pigs, cattle, and chickens from experimental infections. However, the efficacy of these compounds in inducing HDP synthesis varies considerably among distinct compounds. Additionally, the regulation of HDP genes occurs in a gene-specific, cell type–specific, and species-specific manner. In this comprehensive review, we systematically summarized the modulation of HDP synthesis and the mechanism of action attributed to each major class of dietary compounds, including their synergistic combinations, across a spectrum of animal species including humans. We argue that the ability to enhance innate immunity and barrier function without triggering inflammation or microbial resistance positions the nutritional modulation of endogenous HDP synthesis as a promising host-directed approach for mitigating infectious diseases and antimicrobial resistance. These HDP-inducing compounds, particularly in combinations, harbor substantial clinical potential for further exploration in antimicrobial therapies for both human and other animals.

**Statement of Significance**

This review systematically categorizes various dietary compounds capable of inducing host defense peptides by synthesizing a large body of evidence and further summarizes the mechanistic underpinnings of each compound class. This review provides a foundation for future work aimed at developing these compounds as innovative antibiotic alternatives for disease control and prevention, addressing the escalating challenge posed by antimicrobial resistance.

## Introduction

The pervasive and indiscriminate usage of antibiotics in human health care and agriculture has led to the emergence of antibiotic-resistant bacteria that are associated with ∼700,000 annual deaths globally [1,2]. Antibiotic resistance has become a major public health concern, arising from the ability of bacteria to survive and proliferate in the presence of antibiotics. Evolutionarily, bacteria exposed to antibiotics experience selective pressure, allowing resistant strains to thrive and multiply through mutation and horizontal gene transfer [3,4]. Biochemically, bacteria gain resistance through mechanisms such as producing enzymes that degrade antibiotics, altering targets for antibiotics, reducing the influx of antibiotics, and increasing the expression of efflux pumps to expel antibiotics [3,4].

In human medicine, antibiotic resistance complicates the treatment of infections, leading to longer hospital stays, higher medical costs, and increased mortality [5]. In veterinary science, the widespread use of antibiotics in livestock for growth promotion and disease prevention contributes to the emergence of resistant bacteria, which can transfer to humans through direct contact or the food chain [6]. A coordinated One Health approach across human health and veterinary medicine sectors is required to preserve the efficacy of current antimicrobials while novel approaches are developed to control infectious diseases [7]. A pressing need exists for novel approaches in the face of rapid resistance development. Modulation of the synthesis of host defense peptides (HDPs), also known as antimicrobial peptides, has emerged as a promising host-directed antimicrobial strategy [8,9].

HDPs, characterized by their short, positively charged, and amphipathic features, constitute an integral component of innate immunity across a wide spectrum of life forms, ranging from mammals, amphibians, and birds to plants, fungi, and bacteria [10,11]. Two major families of HDPs, namely cathelicidins and defensins (DEFs), are produced in vertebrate animals [[10], [11], [12]]. Cathelicidins are distinguished by the presence of a highly conserved cathelin precursor sequence that undergoes enzymatic cleavage to attain biological activity, while DEFs are defined by the conservation of multiple cysteines in defined positions forming intramolecular disulfide bonds [12]. The synthesis of HDPs is predominantly orchestrated by cell types in direct contact with invading pathogens, such as mucosal epithelial cells, neutrophils, and macrophages [12]. HDPs are either constitutively produced for constant host surveillance and protection or inducibly expressed in response to infection or inflammation [12].

As critical components of innate immunity, HDPs exhibit not only a direct antimicrobial activity against a broad spectrum of pathogens but also multifaceted immunomodulatory properties [[10], [11], [12]]. Their primary antimicrobial mechanism involves membrane disruption through electrostatic interactions with negatively charged microbial membranes, followed by membrane disruption and cell lysis owing to the amphiphilic nature of HDPs [[10], [11], [12]]. Additionally, HDPs play pivotal roles in orchestrating immune responses to pathogen invasion by recruiting different types of immune cells to the sites of infection through direct chemoattraction or stimulation of chemokine secretion [12]. Specific HDPs further contribute to host immune responses to pathogens by promoting phagocytosis or the formation of neutrophil extracellular traps [12]. Moreover, certain HDPs have demonstrated ability to neutralize bacterial endotoxins, modulate host signaling pathways, or promote wound healing to reduce inflammation and facilitate tissue repair [12].

**FIGURE 1:**
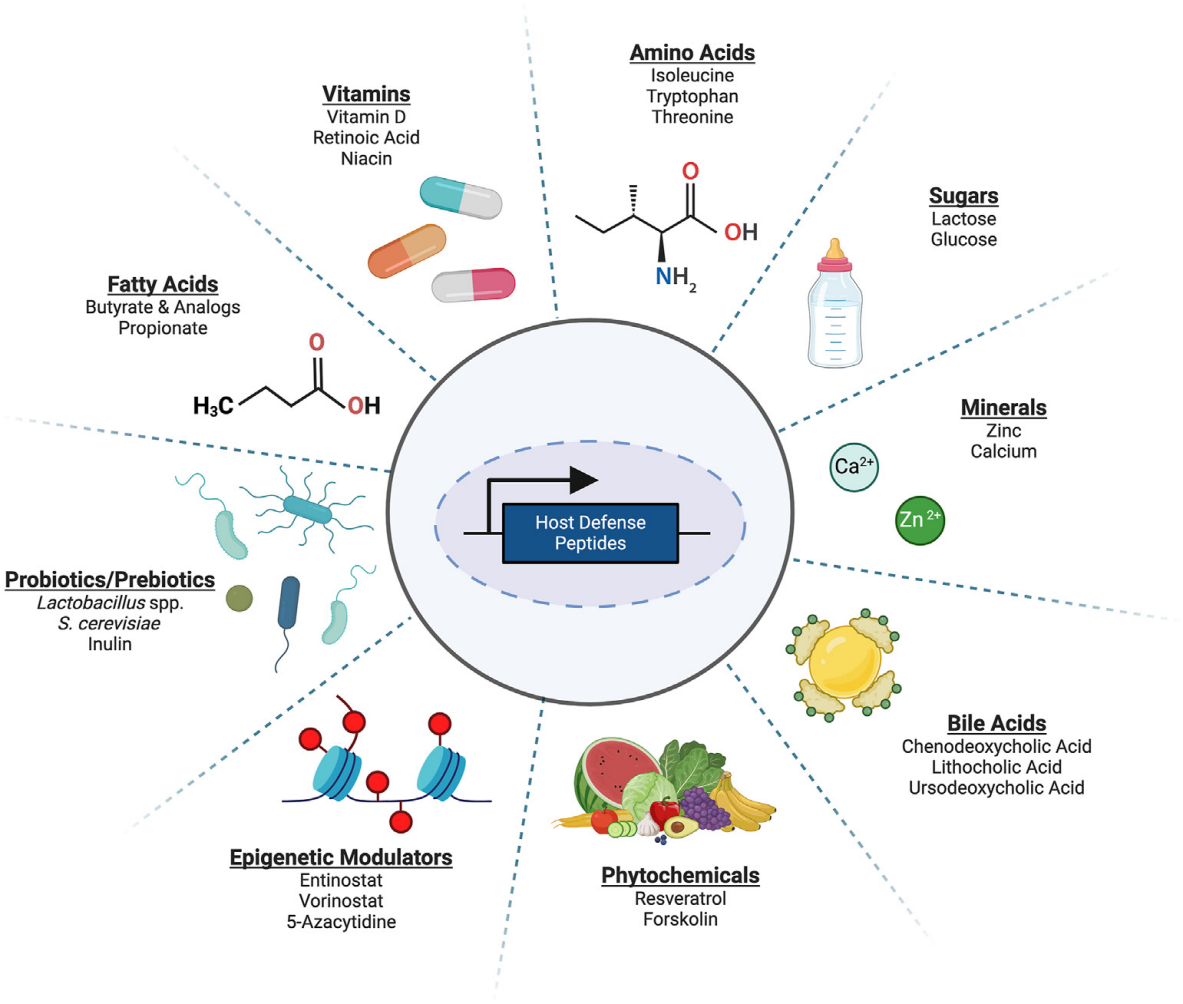
Classification of dietary compounds with the ability to induce host defense peptides. Key examples are listed in each category.

Despite the clinical approval of a few HDPs, such as nisin and daptomycin, challenges persist in the application of the synthetic or recombinant form of HDPs due to high production costs and suboptimal pharmacokinetics [[10], [11], [12]]. Recent years have witnessed a burgeoning interest in identifying nutritional compounds capable of inducing endogenous HDP synthesis in humans and animals. These HDP-inducing nutritional compounds, such as fatty acids, vitamins, animal acids, sugars, minerals, bile acids, phytochemicals, epigenetic modulators, probiotics, and prebiotics, have potential to offer a cost effective alternative approach to disease mitigation [8,9,13] (Figure 1). Importantly, such HDP-inducing compounds pose minimum risk of triggering antimicrobial resistance as they target the host rather than the pathogen. This review aims to systematically summarize current findings regarding the regulation of HDP synthesis by diverse classes of nutritional compounds and the underlying mechanisms. Additionally, the review highlights the potential for synergistic induction of HDPs and improved disease mitigation through a combination of compounds, thereby providing a promising avenue for optimizing host-directed therapeutic strategies.

## Methods

Articles referenced in this systematic review were obtained by searching PubMed for relevant, peer-reviewed, full-text articles that were published in English since the year 1980. The search terms used included (“host defense peptide∗” OR “host defence peptide∗” OR “antimicrobial peptide∗” OR defensin∗ OR cathelicidin∗ OR LL-37) AND (butyrate OR “fatty acid∗” OR vitamin∗ OR “amino acid∗” OR sugar∗ OR mineral∗ OR “bile acid∗” OR polyphenol∗ OR phytochemical∗ OR epigenetic OR probiotic∗ OR prebiotic∗ OR nutrient∗) AND (regulate OR regulation OR modulate OR modulation). References from selected articles were further scanned for relevance and inclusion.

## Results

### Enhancing HDP expression by different classes of dietary compounds

#### Fatty acids

Butyrate, propionate, and acetate are major forms of short-chain fatty acids (SCFAs) that are produced by bacterial fermentation of undigested fiber in the intestine [14]. SCFAs and butyrate in particular are capable of upregulating cathelicidin (*CAMP*) and β-defensin (*DEFB)* expressions in various cell types including human intestinal epithelia [[15], [16], [17]], lung epithelial cells [18,19], and monocytes [20,21] (Table 1). Besides humans, butyrate induces HDP expression in rodents [[22], [23], [24]], chickens [25,26], cattle [27,28], and pigs [[29], [30], [31], [32], [33]], suggesting the conservation of such an innate defense mechanism. Several butyrate analogs, such as 4-phenylbutyrate (PBA), 4-hydroxybutyrate, isobutyrate, benzylbutyrate, trans-cinnamyl butyrate, and glyceryl tributyrate, had a similar or even superior potency than butyrate in HDP induction in humans [20], rabbits [37], rats [39], and pigs [29,38]. Because of rapid absorption and metabolism of butyrate in the gastrointestinal tract, some of these butyrate analogs may have better potential for in vivo applications.

**TABLE 1:** Induction of HDP synthesis by different classes of dietary compounds

Compound	Cells or tissues	HDPs	References
Fatty acids			
Butyrate	Human HT-29, SW620	*CAMP*	[15,16]
	Human HT-29, Caco-2	*DEFB4*	[17]
	Human A549	*DEFB1*	[18]
	Human EBC-1, THP-1	*CAMP*	[[19], [20], [21]]
	C57BL/6 mouse spleen	*Camp*	[22]
	Mouse Paneth cells	*Defa1, Defb1, Reg3g*	[23,24]
	Chicken HD11, HTC, primary monocytes, bone marrow cells, crop, jejunum, and cecum explants	*AvBD3, AvBD4, AvBD5, AvBD8, AvBD9, AvBD10, AvBD14, CATHB1*	[25,26]
	Bovine MEC	*DEFB5, DEFB10, TAP, LAP*	[27]
	Bovine MAC-T	*DEFB5*	[28]
	Porcine IPEC-J2, 3D4/31, primary monocytes	*PBD2, PBD3, PG1-5, PEP2C*	[29]
	Porcine ileum and colon, 3D4/2	*PBD2, PBD3, PG1-5, PMAP37, PR-39*	[30]
	Porcine PK-15	*PBD3, PBD115, PBD123, PBD128, PEP2C*	[31]
	Porcine IPEC-J2, jejunum, cecum	*PBD3, PEP2C*	[32,33]
Free fatty acids and their derivatives	Human HT-29, U937	*CAMP*	[20]
	Human SZ95 sebocytes	*DEFB4*	[34]
	Porcine IPEC-J2	*PBD2, PBD3, PEP2C*	[29]
	Chicken HD11, primary monocytes	*AvBD9*	[35]
4-Phenylbutyrate	Human VA10, U937, A498, HT-29	*CAMP*	[36]
	Human VA10	*DEFB1*	[36]
	Rabbit lung and rectum epithelium	*CAMP*	[37]
	Porcine IPEC-J2	*PBD1, PBD3, PEP2C*	[38]
4-Hydroxybutyrate	Rat bone marrow–derived macrophages	*Camp, Defb4*	[39]
Isobutyrate, propionate	Human SW620 colonocytes	*CAMP*	[15]
Valproic acid	Human A549	*DEFB1*	[18]
Caprylic acid, nonanoic acid	Porcine IPEC-J2	*PBD1, PBD2*	[40]
Oleic acid	Mouse hair follicle sebaceous glands	*Defb1*	[34]
Propionate, hexanoate	Bovine MEC	*TAP*	[41]
Vitamins			
Vitamin D-3	Human keratinocytes, monocytes, neutrophils, neutrophil progenitors, SCC25, Calu-3, U937, HL60, bone marrow cells, HaCat, HT-29, airway epithelial organoids	*CAMP*	[[42], [43], [44], [45], [46], [47]]
	Human SCC25, Calu-3, primary keratinocytes	*DEFB4*	[42]
	Murine intestinal organoids	*Defa5, Reg3g*	[48]
	Chicken embryonic intestinal epithelial cells and PBMCs	Multiple β-defensins	[49]
	Bovine MEC	*DEFB1, LAP, S100A7*	[50]
	Bovine peripheral blood leukocytes	*DEFB3, DEFB7, DEFB8, DEFB10, LAP, S100A8*	[51]
Calcipotriol	Human skin biopsies	*CAMP*	[52]
Retinoic acid	Human Bronchial Epithelial Cells	*DEFB3, DEFB4, CAMP*	[53]
	Human U937	*CAMP*	[54]
	Murine intestinal organoids	*Defa5, Reg3g*	[48]
	Mouse Preadipocytes 3T3-L1	*Camp*	[55]
Retinol, retinaldehyde	Mouse skin	*DEFB3*	[56]
Nicotinamide	Human neutrophils	*CAMP*	[57]
	Mouse bone marrow cells	*Camp*	[57]
Niacin	Piglet ileum, jejunum, colon	*PBD2, PG1-5, PR39*	[58,59]
Vitamin C	Human keratinocytes	*DEFB1*	[60]
	Digestive gland of *Haliotis discus hannai*	*DEFB*	[61]
Amino acids			
Isoleucine	Human HCT-116 colon cells	*DEFB1*	[62]
	Human A549 lung epithelial cells	*DEFB4*	[63]
	BALB/c mouse lung	*DEFB3, DEFB4*	[63]
	Bovine MDBK kidney cells	Bovine β-defensins	[64]
	Porcine IPEC-J2	*PBD1, PBD2, PBD3*	[65]
	Porcine IPEC-J2, jejunum and ileum	*PBD1, PBD2, PBD114, PBD129*	[66]
	Intestine of *P. vachelli* × *L. longirostris*	*DEFB, HAMP*	[67]
Leucine	Piglet jejunum and ileum, IPEC-J2	*PBD1, PBD2, PBD114, PBD129*	[66]
	Intestine of *P. vachelli* × *L. longirostris*	*DEFB*	[68]
Valine	Piglet jejunum and ileum, IPEC-J2	*PBD1, PBD2, PBD114, PBD129*	[66]
Tryptophan	Mouse ileal mucosa	*Reg3g, Reg3b*	[69]
	Rat jejunal and ileal mucosa	*Defb2*	[70]
	Porcine IPEC-J2	*PBD1, PBD2, PBD3, PBD115, PG1-5, PR-39*	[70]
	Piglet jejunal, ileal, colon mucosa	*PBD2*	[71]
Threonine	Porcine IPEC-J2	*PBD1, PBD2, PBD3*	[72]
	Head kidney of *Ctenopharyngodon idella*	*DEFB1, HAMP, LEAP-2B*	[73]
	Spleen of *Ctenopharyngodon idella*	*DEFB1, HAMP, LEAP-2A, LEAP-2B*	[73]
Arginine	Human HCT-116 colon cells	*DEFB1*	[62]
	Mouse Paneth cells	*Defa29*	[74]
Glutamine	Mouse Paneth cells	*Reg3γ*	[74]
	Mouse ileum	*Defa4*	[75]
Sugars			
Lactose	Human HT-29, Caco-2, T84, THP-1	*CAMP*	[76]
	Chicken HD11 macrophages	*AvBD9, AvBD14*	[77]
	Piglet jejunal mucosa	*PBD1, PBD2, PBD3*	[78]
Glucose	Human NHK keratinocytes	*DEFB1*, *CAMP*	[60]
	Human HEK-293, HCT-116	*DEFB1*	[79]
	Rat kidney	*DEFB1*	[80]
	Chicken HD11 macrophages	*AvBD9, AvBD14*	[77]
Maltose, trehalose	Human HT-29 colon epithelia	*CAMP*	[76]
Galactose	Chicken HD11 macrophages	*AvBD9, CATHB1, AvBD14*	[77]
Trehalose	Chicken HD11 macrophages	*AvBD9, CATHB1*	[77]
Maltose, sucrose, fructose	Chicken HD11 macrophages	*AvBD9*	[77]
Minerals			
Zinc	Human Caco-2	*CAMP*	[81]
	Porcine IPEC-J2	*PBD1, PBD2, PBD3*	[65]
Zinc gluconate	Human skin explants	*DEFB4,* Psoriasin	[82]
Calcium	Primary human keratinocytes	*DEFB3, DEFB4, DEFB104*	[83]
	Human HaCaT, PHK16-0b	*DEFB1, DEFB3*	[84]
Bile acids			
LCA	Human HT-29, keratinocytes	*CAMP*	[16,85]
CDCA, UDCA	Human biliary epithelial cells	*CAMP*	[86]
TMCA, THDCA	Mouse 3T3-L1 adipocytes	*Camp*	[87]
Phytochemicals			
EGCG	Human B11 gingival epithelial	*DEFB1, DEFB4*	[88]
	Human BEAS-2B	*DEFB3*	[89]
	Porcine IPEC-J2	*PBD1, PBD2*	[90]
	Chicken PBMCs	*AvBD9*	[91]
Quercetin	Human HepG2 hepatocytes, rat liver	*HAMP*	[92]
	Chicken HTC, PBMCs	*AvBD4-7, AvBD9, AvBD14*	[91]
	Chicken ileum	Multiple β-defensins	[93]
	Zebrafish liver	Defensin*, LEAP2*	[94]
Saponarin	Human HaCaT keratinocytes	*CAMP*	[95]
Apigenin	Human HaCaT keratinocytes	*CAMP, DEFB1, DEFB3, DEFB4*	[96]
Genistein	Human keratinocytes	*CAMP*	[97]
Xanthohumol	Porcine 3D4/31 macrophages	*PBD3, PEP2C, PG1-5*	[98]
	Porcine jejunal explants	*PBD3, PG1-5*	[98]
Calycosin	Porcine IPEC-J2	*PBD2, PBD3, PEP2C*	[98]
Datiscetin	Chicken HTC, jejunal explants	*AvBD9*	[99]
Isoloquiritigenin	Human Caco-2 colonic epithelial	*DEFB3*	[100]
	Porcine IPEC-J2	*PBD3, PEP2C, PG1-5*	[98]
Resveratrol	Human HepG2 hepatocytes, rat liver	*HAMP*	[92]
	Human periodontal ligament	*DEFB4, DEFB3*	[101]
	Human HaCaT keratinocytes, U937	*CAMP*	[102,103]
	Mouse ileum	*Defa3, Defa5, Defa20*	[104]
	Rat liver	*Hamp*	[92]
	Chicken PBMCs	*AvBD9*	[91]
Polydatin	Human HaCat keratinocytes	*DEFB4*	[105]
Pterostilbene	Human U937 monocytes	*CAMP*	[103]
	Porcine 3D4/31 macrophages	*PBD3, PG1-5*	[98]
Isorhapontigenin	Porcine IPEC-J2 intestinal epithelial	*PBD3, PEP2C, PG1-5*	[98]
	Porcine jejunal explants	*PBD3, PG1-5*	[98]
Ellagic acid	Human gingival epithelial	*DEFB4*	[106]
Caffeic acid	Mouse tongue	*Defb3*	[107]
Anacardic acid	Chicken PBMCs	*AvBD9*	[91]
Curcumin	Human U937 monocytes, HT-29	*CAMP*	[108]
	Grass carp liver, PBMCs	*HAMP, LEAP-2, DEFB*	[109]
Forskolin	Human HT-29, Caco-2, HaCaT, INT407, A549	*CAMP*	[110]
	Chicken HD11, HTC macrophages	*AvBD9*	[26]
Andrographolide	Human Caco-2 colonic epithelial	*DEFB3*	[100]
Oridonin	Human Caco-2 colonic epithelial	*DEFB3*	[100]
Tetrandrine	Chicken HTC cells, jejunal explants	*AvBD9*	[99]
Sanguinarine	Chicken HTC cells, jejunal explants	*AvBD9*	[99]
Deoxyshikonin	Porcine IPEC-J2, 3D4/31 cells	*PBD3, PEP2C, PG1-5*	[98]
	Porcine jejunal explants	*PBD3, PG1-5*	[98]
Epigenetic modulators			
Trichostatin A	Human SW620 colon epithelial	*CAMP*	[15]
	Human A549 lung epithelial, human NCI-H727 lung epithelial	*DEFB1*	[18]
	Human primary gingival epithelial	*DEFB4*	[111]
	Human Caco-2 colonic epithelial, primary colonic	*CAMP, DEFB3, DEFB4*	[112]
	Human VK2/E6E7 vaginal keratinocytes	*DEFB1*	[113]
	Chicken HTC macrophages	*AvBD9*	[99]
	Porcine IPEC-J2 intestinal epithelial	*PBD3*	[114]
	Primary bovine mammary epithelial	*DEFB3, DEFB4, DEFB7, DEFB10*	[115]
	Rat testis, cauda	*Defb1, Defb2*	[116]
Vorinostat	Human THP-1 monocytes	*DEFA1, DEFA5, DEFA6, DEFB4*	[117]
	Human Caco-2/TC7 cells	*DEFB4*	[118]
	Human Caco-2 and primary colonic epithelial cells	*CAMP, DEFB3, DEFB4*	[112]
	Human Huh7 hepatocytes	*LEAP-1*	[119]
	Porcine IPEC-J2 intestinal epithelial, porcine 3D4/31 macrophages	*PBD2, PBD3*	[114]
	Chicken HTC macrophages	*AvBD4, AvBD8, AvBD9, AvBD10, AvBD14*	[120]
Entinostat	Human HT-29 colonic epithelial	*CAMP*	[121,122]
	Rabbit ileal and rectal epithelium	CAMP	[121,123]
	Chicken crop and jejunum	*AvBD9, AvBD10, AvBD14, CATHB1*	[124]
Aroylated phenylenediamines	Human HT-29 colonic epithelial	*CAMP*	[121]
ADP HO53	Human BCi and VA10 bronchial epithelial cells	*CAMP, DEFB1*	[125]
ADP HO56	Human BCi and VA10 bronchial epithelial cells	*CAMP, DEFB1*	[125]
Mocetinostat	Porcine IPEC-J2 intestinal epithelial	*PBD3*	[114]
	Chicken HTC macrophages	Multiple β-defensins	[126]
	Chicken jejunal explants	*AvBD9*	[126]
	Chicken HTC and HD11 macrophages	*AvBD9*	[120]
RGFP966	Human Huh7 hepatocytes	*LEAP-1*	[119]
Panobinostat	Porcine IPEC-J2 intestinal epithelial	*PBD2, PBD3, PG1-5*	[114]
LAQ824	Porcine IPEC-J2 intestinal epithelial	*PBD2, PBD3, PG1-5*	[114]
SB939	Porcine IPEC-J2 intestinal epithelial	*PBD2, PBD3, PG1-5*	[114]
Apicidin	Human A549 lung epithelial	*DEFB1*	[18]
	Chicken HTC macrophages	*AvBD9*	[99]
	Porcine IPEC-J2 intestinal epithelial	*PBD2, PBD3*	[114]
Depudecin	Human A549 lung epithelial	*DEFB1*	[18]
	Chicken HTC macrophages	*AvBD9*	[99]
	Porcine IPEC-J2 intestinal epithelial	*PBD3*	[114]
SGI-1027	Chicken HTC macrophages	*AvBD9*	[120]
BIX01294	Chicken HTC macrophages	*AvBD9*	[120]
UNC1999	Chicken HTC macrophages	*AvBD9*	[120]
5-Azacytidine	Human gingival epithelial cells	*DEFB4*	[111]
	Human OSC-19, BSC-OF, SAS, HSC-2, HSC-4, HSY oral squamous	*DEFB4*	[127]
	Human HSC-3 and HSC-4 oral squamous, HaCaT keratinocytes, TR146 buccal epithelial	LL-37	[128]
	Human VK2/E6E7 keratinocytes	*DEFB1*	[113]
	Human HC-OA chondrocytes	*CAMP*	[129]
	Bovine mammary epithelial	*DEFB3, DEFB5, DEFB10, EBD, LAP, TAP*	[115]
	*A. pernyi* larvae	Attacin, cercropin, and lebocin	[130]
	Rat cauda	*Defb1, Defb2, Defb27*	[116]
	Rat testis	*Defb1, Defb2, Defb30*	[116]
	Rat caput	*Defb30, Defb36*	[116]
Probiotics			
*Lactobacillus gasseri*	Human VK2/E6E7 keratinocytes	*DEFB1*	[113]
*Lactobacillus fermentum* K11-Lb3 and K2-Lb6	Human Caco-2 colonic epithelial	*DEFB4*	[131]
*Ligiactobacillus salivarius* SMXD51	Human Caco-2/TC7 cells	*DEFB4*	[132]
*Lacticaseibacillus paracasei* CBA L74	Human Caco-2 colonic epithelial	*CAMP, DEFB4*	[133]
*Lactobacillus helveticus SBT2171*	Human Caco-2 colonic epithelial, HSC-4 tongue epithelial	*DEFB4*	[134]
	Human Ca9-22 gingival cells	*DEFB3, DEFB4*	[135]
	Mouse gingival tissue	*Defb4, Defb14*	[135]
*Lactobacillus rhamnosus GG*	Human SW480 colonic epithelial	*DEFB4*	[136]
*Bifidobacterium longum*	Human SW480 colonic epithelial	*DEFB4*	[136]
*L. paracasei* SD1, L. rhamnosus SD4, *L. fermentum* SD7, *L. rhamnosus* SD11, *L. rhamnosus* GG	Primary human gingival epithelial cells	*DEFB4, DEFB104*	[137]
*L. rhamnosus* Lcr35	Human VK2/E6E7 vaginal epithelia	*DEFB4*	[138]
*Lactobacillus crispatus*	Human HeLa cells	*DEFB3, DEFB4*	[139]
*Lactobacillus johnsonii* NCC 533	Human epidermis	*CAMP, DEFB1, DEFB4, DEFB103B*	[140]
*Lactobacillus delbrueckii* subsp. bulgaricus 8481	Human serum	*DEFB4*	[141]
BB12	Human infant stool samples	*CAMP, DEFB4*	[142]
*Escherichia coli* Nissle 1917, *Pediococcus pentosaceus*, *Lactobacillus acidophilus*, *L. fermentum*	Human Caco-2 colonic epithelial	*DEFB4*	[143]
*Lactobacillus reuteri* 5454, B. animalis ssp.	Mouse ileum	*Reg3b, Reg3g*	[144]
*L. paracasei* subsp. paracasei CNCM I-1518	Rat ileum	*Defb1*	[145]
*Lactobacillus casei* CRF28, UW1	C57BL/6 mouse ileum	*Defa-rs1*	[146]
*L. casei* 32G, CRF28, BL23, M36	C57BL/6 mouse ileum	*Reg3β*	[146]
*Lactobacillus plantarum ZLP001*	Piglet jejunum, ileum, IPEC-J2, 3D4/31	*PBD2, PBD3, PBD114, PBD129, PG 1–5, PEP2C*	[147,148]
*Lactobacillus amylovorus* SLZX20-1	Porcine IPEC-J2	*PBD1, PEP2C, PG1-5*	[149]
*Bacillus subtilis* CP9	Porcine IPEC-J2	*PG1*	[150]
*B. subtilis* and *L. salivarius*	Piglet duodenum	*PBD2*	[151]
*L. rhamnosus GG*	Piglet jejunum	*PBD1, PMAP-37*	[152]
*L. reuteri* *Clostridium butyricum*	Chicken cecum	*AvBD4* *AvBD1, CATH3*	[153]
*L. casei* BL23	Bovine MEC	*TAP*	[154]
*Saccharomyces cerevisiae CNCM I-1077*	Bovine rumen and colon epithelium	*DEFB1*	[155]
Prebiotics			
Inulin	Mouse Paneth cells	*Defb1, Defa1, Defa4, Defa29,* Defa5, *Reg3g*	[24]
Long-chain inulin-type fructans	Mouse colon	*Defb1, Camp*	[156]
*Dendrobium huoshanense* polysaccharides	Mouse jejunum and ileum	Total β-defensin protein	[157]

Abbreviations: AvBD, avian β-defensin; CAMP, cathelicidin; CDCA, chenodeoxycholic acid; DEFA, α-defensin; DEFB,β-defensin; EGCG, epigallocatechin gallate; HAMP, hepcidin antimicrobial peptide; HDP, host defense peptide; LAP, lingual antimicrobial peptide; LCA, lithocholic acid; LEAP, liver-enriched antimicrobial peptide; PBD, porcine β-defensin; Reg3, regenerating islet-derived protein 3; TAP, tracheal antimicrobial peptide; TDCA, taurodeoxycholic acid; THDCA, taurohyodeoxycholic acid; TMCA, α-tauromuricholic acid; UDCA, ursodeoxycholic acid.

Consistent with their HDP-inducing activity in cells, oral supplementation of sodium butyrate or PBA improved the clinical outcomes of shigellosis in both humans and rabbits by counteracting *Shigella*-induced downregulation of *CAMP* expression in the intestinal tract [37,158]. In chickens, dietary supplementation of butyrate significantly reduced *Salmonella* sp. colonization in the cecum of experimentally infected chickens [25]. Additionally, oral supplementation with sodium butyrate reduced the *Corynebacterium pseudotuberculosis* load, enhanced *Camp* expression, and alleviated lesions in the spleens of infected mice [22]. Similarly, butyrate, caprylic acid, and nonanoic acid reduced the bacterial load, mitigated intestinal inflammation, and upregulated HDP expressions in piglets challenged with *Escherichia coli* 0157:H7 [30,40]. A recent study also showed the ability of butyrate to counteract deoxynivalenol-mediated suppression of intestinal HDPs in weaned piglets [33].

Comparison of the HDP-inducing activity among free saturated fatty acids of 1–18 hydrocarbons in humans, pigs, and chickens revealed that SCFAs are the most potent, whereas fatty acids with longer aliphatic chains quickly lose HDP-inducing potency [20,29,39]. However, relative HDP-inducing potency of individual fatty acids varies among animal species. Butyrate appears to be the most efficacious HDP inducer in chicken and porcine epithelial and monocytic cells [29,35], whereas valerate, hexanoate, and heptanoate are capable of triggering stronger HDP expression than butyrate when used at higher concentrations in humans [20]. It will be interesting to evaluate the in vivo efficacy of valerate, hexanoate, and heptanoate and their analogs in HDP induction and bacterial clearance in humans.

It is abundantly clear that different HDPs are regulated differently by fatty acids. For example, PBA stimulated human *CAMP* and *DEFB1* expression but not *DEFB3, DEFB4*, and *DEFB104* in human lung epithelial cells [36]. Lauric acid, palmitic acid, and oleic acid upregulated *DEFB4* but not *CAMP* or *DEFB1* in human sebocytes [34]. In mice, oleic acid preferentially promoted the induction of mouse *Defb2* in the hair follicle sebaceous glands of mouse ear skin [34]. Propionate and hexanoate potentiated bovine tracheal antimicrobial peptide expression but not *DEFB5* [41].

**FIGURE 2:**
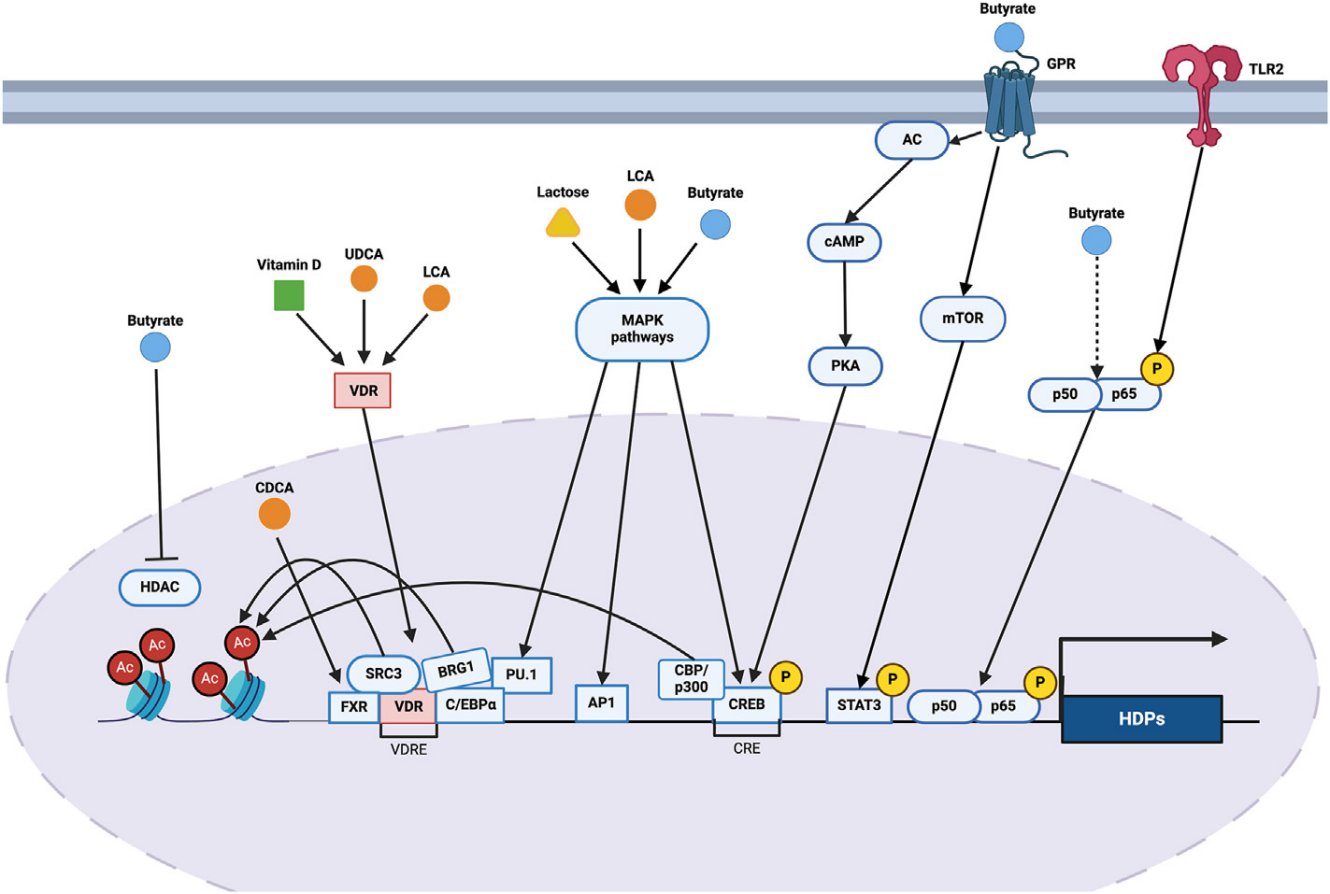
Molecular mechanisms of HDP induction by butyrate, vitamin D-3, bile acids, and lactose. An arrowhead indicates stimulation of a pathway, while a T-bar indicates inhibition of a pathway. See text for details. AC, acetyl group; AP1, activator protein-1; *BRG1*, Brahma-related gene 1; CDCA, chenodeoxycholic acid; C/EBPα, CCAAT enhancer binding protein α; CRE, cAMP-response element; CREB, cAMP-response element-binding protein; FXR, farnesoid X receptor; GPR, G protein-coupled receptors; HDAC, histone deacetylase; HDP, host defense peptide; LCA, lithocholic acid; MAPK, mitogen-activated protein kinases; mTOR, mammalian target of rapamycin; p50/p65, NF-κB proteins p50 and p65 heterodimer; PU.1, an ETS-family transcription factor; RXR, retinoid X receptor; SRC, steroid receptor coactivator; STAT, signal transducer and activator of transcription; TLR, toll-like receptor; UDCA, ursodeoxycholic acid; VDR; vitamin D receptor.

A growing body of evidence has suggested that SCFAs and butyrate in particular induce HDP expression primarily by acting as histone deacetylase (HDAC) inhibitors [159]. HDAC1, HDAC2, and HDAC3 were downregulated by butyrate in human THP-1 monocytes [21], associated with an increase in histone acetylation along the promoters of human, bovine, and porcine HDPs, which is associated with active gene transcription [19,28,30]. Free fatty acid receptors, such as GPR41/FFAR3, GPR43/FFAR2, and GPR109a/HCAR2, are also involved in SCFA-mediated HDP induction, leading to downstream activation of the mammalian target of rapamycin and signal transducers and activator of transcription (STAT) 3 [23,160] (Figure 2).

Additionally, Toll-like receptor (TLR) 2–mediated NF-κB activation is required for butyrate-induced expression of porcine β-defensins (PBDs) in porcine kidney and intestinal cells, while HDAC inhibition is insufficient [31,32,38]. NF-κB signaling is also required for butyrate-induced *DEFB2* production by human colonic epithelial cells [118]. In fact, TLR2 expression is enhanced in bovine epithelial cells treated with butyrate, while concurrent p38 phosphorylation implicates the involvement of the mitogen-activated protein kinase (MAPK) pathway [27,28]. Canonical MAPK family members including MEK, extracellular signal-regulated kinase (ERK), and JNK regulate butyrate-induced *CAMP* expression in human colon, gastric, and bronchial cell lines but not in heptocarcinoma cell lines [15,36]. Furthermore, butyrate-enhanced *CAMP* expression in human colonic epithelial cells is mediated by PU.1, a transcription factor activated by MAPK [16]. Additional transcription factors, such as activator protein (AP) 1 and cAMP (cyclic adenosine monophosphate)-response element-binding protein (CREB), are key regulators in butyrate-induced human *CAMP* and *DEFB4* expression [19,110,161].

#### Vitamins

Vitamin D-3 is a potent HDP inducer in several human cell types including epithelial cells, keratinocytes, monocytes, and neutrophils, leading to an increase in the antimicrobial activity of the host cells [[42], [43], [44], [45]]. For example, the growth of *Mycobacterium tuberculosis* was significantly suppressed in macrophages exposed to 1,25-dihydroxyvitamin D-3, also known as calcitriol [46,162] (Table 1 [[15], [16], [17], [18], [19], [20], [21], [22], [23], [24], [25], [26], [27], [28], [29], [30], [31], [32], [33], [34], [35], [36], [37], [38], [39], [40], [41], [42], [43], [44], [45], [46], [47], [48], [49], [50], [51], [52], [53], [54], [55], [56], [57], [58], [59], [60], [61], [62], [63], [64], [65], [66], [67], [68], [69], [70], [71], [72], [73], [74], [75], [76], [77], [78], [79], [80], [81], [82], [83], [84], [85], [86], [87], [88], [89], [90], [91], [92], [93], [94], [95], [96], [97], [98], [99], [100], [101], [102], [103], [104], [105], [106], [107], [108], [109], [110], [111], [112], [113], [114], [115], [116], [117], [118], [119], [120], [121], [122], [123], [124], [125], [126], [127], [128], [129], [130], [131], [132], [133], [134], [135], [136], [137], [138], [139], [140], [141], [142], [143], [144], [145], [146], [147], [148], [149], [150], [151], [152], [153], [154], [155], [156], [157]]). The positive influence of treatment with vitamin D-3 or its analogs on HDP production was clearly demonstrated in vivo as well. Topical treatment with vitamin D-3 resulted in the upregulation of human *CAMP* in acute skin injuries [52]. Similarly, psoriatic skin biopsies treated with vitamin D-3 had elevated concentrations of LL-37 and reduced proinflammatory cytokines IL-12/23 p40*,* IL-1α*,* IL-1β*,* and TNF-α [163].

Conversely, a deficiency in vitamin D-3 is often associated with an increased susceptibility to infections, likely due to decreased synthesis of HDPs [164]. For instance, patients who experience severe congenital neutropenia, because of a lack of vitamin D-3, experience recurrent infections, whereas patients given vitamin D-3 show restored LL-37 concentrations and reduced infections [47]. In patients with atopic dermatitis, downregulation of serum 25-hydroxyvitamin D-3 and LL-37 concentrations was observed, with a reciprocal increase in the expression of proinflammatory cytokines [165]. In patients with cystic fibrosis, serum vitamin D concentrations are often deficient, perhaps contributing to the frequency of lung infections [166]. Although serum vitamin D concentrations are positively correlated with tuberculosis disease risk [167], a recent clinical trial with 8851 children with latent tuberculosis infection found no significant improvement in disease risk when a weekly dose of 14,000 IU of vitamin D-3 was given for 3 y [168].

In addition to humans, vitamin D-3 enhances the expression of HDPs in pigs [169], chickens [49], and cattle [50,51]. Administration of vitamin D-3 to cattle reduced internalization of *Staphylococcus aureus* in bovine mammary epithelial cells by inducing the expression of lingual antimicrobial peptide, *DEFB10*, and psoriasin [50]. Similarly, an increase in expression of HDPs after vitamin D-3 supplementation was accompanied by enhanced clearance of *Mycobacterium bovis* Bacillus Calmette-Guérin (BCG) vaccine in bovine peripheral blood leukocytes [51]. It is noted that there is clearly a species-specific difference in the HDP-inducing ability of vitamin D-3. Although it is a strong inducer of cathelicidins and several DEFBs in humans and cattle, vitamin D-3 fails to do so in mice, dogs, and sheep, which is likely due to an absence of the vitamin D response (VDR) elements in the promoter of HDP genes in those species [43,170]. However, intestinal α-defensins (DEFAs) appear to be regulated by vitamin D-3 in mice [48].

Vitamin D-3 induces *CAMP* and *DEFB4* gene expression by binding to VDR to activate respective gene promoters containing VDR elements in different human cell types [42,43] (Figure 2). More recently, transcription factors PU.1 and C/EBPα were found to participate in vitamin D-3 regulation of *CAMP* and *DEFB4* gene expression [171]. Binding of PU.1 and C/EBPα recruits Brahma-related gene 1, a component of the SWI/SNF chromatin remodeling complex, to the *CAMP* gene promoter, leading to an increase in H4 acetylation [171]. Additionally, steroid receptor activator 3, which has intrinsic histone acetyltransferase activity, is critical for vitamin D-3–mediated induction of *CAMP*, as steroid receptor activator 3 knockdown prevented the upregulation of the *CAMP* gene [45].

Retinoic acid, a metabolite of vitamin A, has conflicting effects on HDP induction. Retinoic acid induces *CAMP*, *DEFB3*, and *DEFB4* gene expression in human bronchial epithelial cells [53] but inhibits *DEFB3*, *DEFB4*, and *DEFB104* in primary keratinocytes in response to proinflammatory cytokines, bacteria, phorbol myristate acetate, or calcium [83]. Although retinoic acid induces *CAMP* in human U937 monocytes, no change is seen in K562 lymphoblasts [54]. In mice, retinoic acid upregulated *Defa1*, *Defa5*, *Defa21*, and regenerating islet-derived protein 3-γ (*Reg3g*) in intestinal organoids [48], *Camp* in primary preadipocytes [55], along with *Reg3b* and *Reg3g* in lymphocytes [172]. Retinol and retinaldehyde significantly increased *Defb3* expression in mouse skin, suggesting that topical retinols may be a potential treatment of cutaneous infections via increased expression of HDPs [56]. The expression of porcine cathelicidin *PR-39* was enhanced in bone marrow cells by retinoic acid [173]. However, retinoic acid failed to induce HDP expression in ovine respiratory epithelial cells [174].

Niacin, also known as vitamin B-3, was found to elevate HDP gene expression in porcine IPEC-J2 cells as well as throughout the intestinal tract of piglets [58,59]. Notably, supplementation of niacin alleviated the disease severity of porcine deltacoronavirus [58] as well as enterotoxigenic *E*. *coli* K88 in weaned piglets [59]. Niacin appeared to induce porcine HDP gene expression through epigenetic modifications. Improved HDP mRNA concentrations coincided with increased expression concentrations of sirtuin (SIRT) 1 and reduced HDAC7 in the intestine of piglets in response to niacin supplementation [59]. Niacin enhanced phosphorylation of histone H3 at Ser10 (H3S10p) in the ileum as well as acetylation of lysine 9 on histone 3 (H3K9ac) and H3K27ac in the colon of piglets challenged with ETEC K88 [59].

Nicotinamide, an amide form of niacin, improved the expression of *CAMP* in human neutrophils but not in monocytes [57]. Increased Camp protein concentrations were also detected in bone marrow mononuclear cells of mice supplemented with nicotinamide, while the treatment enhanced *S*. *aureus* killing by ≤1000-fold in a systemic murine infection model [57]. Vitamin C, also known as ascorbic acid, increased *DEFB1* but not *CAMP* gene expression in human keratinocytes, [60]. Vitamin C was similarly found to increase *DEFB* mRNA concentrations in the digestive gland of abalone [61].

#### Amino acids

Essential, branched-chain amino acids, including isoleucine, leucine, and valine, are each capable of upregulating HDP expression. Isoleucine increased DEFBs across multiple animal species, including humans [62,63], mice [63], cattle [64], pigs [65,175], and catfish [67] (Table 1 [[15], [16], [17], [18], [19], [20], [21], [22], [23], [24], [25], [26], [27], [28], [29], [30], [31], [32], [33], [34], [35], [36], [37], [38], [39], [40], [41], [42], [43], [44], [45], [46], [47], [48], [49], [50], [51], [52], [53], [54], [55], [56], [57], [58], [59], [60], [61], [62], [63], [64], [65], [66], [67], [68], [69], [70], [71], [72], [73], [74], [75], [76], [77], [78], [79], [80], [81], [82], [83], [84], [85], [86], [87], [88], [89], [90], [91], [92], [93], [94], [95], [96], [97], [98], [99], [100], [101], [102], [103], [104], [105], [106], [107], [108], [109], [110], [111], [112], [113], [114], [115], [116], [117], [118], [119], [120], [121], [122], [123], [124], [125], [126], [127], [128], [129], [130], [131], [132], [133], [134], [135], [136], [137], [138], [139], [140], [141], [142], [143], [144], [145], [146], [147], [148], [149], [150], [151], [152], [153], [154], [155], [156], [157]]). For example, isoleucine supplementation increased *Defb3* and *Defb4* expression, which led to a decrease in intestinal bacillary loads and tissue damage after mice were inoculated with a multidrug resistant *M*. *tuberculosis* H37Rv [63]. Leucine increased *Defa1* in mouse Paneth cells [23], 4 DEFBs in the jejunum and ileum of piglets [66], as well as *DEFB* transcription in the intestine of hybrid catfish [68]. Valine supplementation improved intestinal DEFB mRNA concentrations in piglets [66] and upregulated *DEFB1* and *CATH7* in cultured mammary epithelial cells of goats [176].

Tryptophan and threonine are essential amino acids to mammals that have also been shown to upregulate HDP production. Tryptophan improved both mRNA and protein concentrations of Defb2 in the jejunal and ileal mucosa of rats [177], as well as *Reg3g* and *Reg3b* mRNA concentrations in mouse ileal mucosa [69]. Tryptophan also increased the expression of multiple HDP genes in porcine IPEC-J2 cells [70]. Furthermore, diets with adequate tryptophan (0.21%, 0.28%, or 0.35%) elevated PBD2 protein concentration across the small intestine of piglets compared with a 0.14% tryptophan diet [71]. L-threonine similarly induced the transcription of DEFBs in porcine IPEC-J2 cells [72]. Supplementation with threonine compared with a threonine-deficient diet enhanced mRNA concentrations of hepcidin antimicrobial peptide (*HAMP*), liver-enriched antimicrobial peptide (*LEAP*) 2, and *DEFB1* in the head kidney and spleen of grass carp [73].

Furthermore, arginine upregulated *DEFB1* in human HCT-116 colonic epithelial cells [62,74]. Dietary arginine enhanced *Defa29* and *Reg3g* expression in mouse Paneth cells and decreased *E. coli* colonization in the jejunum of challenged mice [74]. In porcine IPEC-J2 cells, L-arginine triggered DEFB transcription and could ameliorate LPS-induced inflammation [178]. Glutamine is a non-essential amino acid capable of HDP induction in mice. *Defa4* mRNA was increased in the small intestine of mice supplemented with glutamine [75]. Similarly, glutamine augmented *Defa29* and *Reg3g* mRNA expression and decreased *E. coli* colonization in mice [74].

Isoleucine, tryptophan, and threonine involve SIRT1 to initiate a signaling cascade that enhances HDP gene expression; however, whether SIRT1 is activated or inhibited varies among individual amino acids. In porcine intestinal epithelial cells and the intestine of hybrid catfish, isoleucine activated a SIRT1/ERK/90RSK signaling pathway to upregulate several HDP genes [66,67]. However, threonine suppressed *SIRT1* expression in porcine intestinal epithelial cells, which enhanced acetylation of p65, promoted translocation of p65 into the nucleus, and activated NF-κB [72]. Tryptophan, on the contrary, had no direct effect on *SIRT1* expression but suppressed LPS-induced SIRT1 expression in mouse ileal mucosa and porcine intestinal epithelial cells [69]. The mammalian target of rapamycin pathway is involved in induction of DEFBs by tryptophan [177] and arginine [178].

#### Sugars

Lactose, a disaccharide sugar, was shown to induce *CAMP* transcription in a dose-dependent and time-dependent manner in human HT-29 and T84 colonic epithelial cells and THP-1 monocytes/macrophages [76] (Table 1 [[15], [16], [17], [18], [19], [20], [21], [22], [23], [24], [25], [26], [27], [28], [29], [30], [31], [32], [33], [34], [35], [36], [37], [38], [39], [40], [41], [42], [43], [44], [45], [46], [47], [48], [49], [50], [51], [52], [53], [54], [55], [56], [57], [58], [59], [60], [61], [62], [63], [64], [65], [66], [67], [68], [69], [70], [71], [72], [73], [74], [75], [76], [77], [78], [79], [80], [81], [82], [83], [84], [85], [86], [87], [88], [89], [90], [91], [92], [93], [94], [95], [96], [97], [98], [99], [100], [101], [102], [103], [104], [105], [106], [107], [108], [109], [110], [111], [112], [113], [114], [115], [116], [117], [118], [119], [120], [121], [122], [123], [124], [125], [126], [127], [128], [129], [130], [131], [132], [133], [134], [135], [136], [137], [138], [139], [140], [141], [142], [143], [144], [145], [146], [147], [148], [149], [150], [151], [152], [153], [154], [155], [156], [157]]). In fact, several other monosaccharide and disaccharide sugars such as glucose, galactose, trehalose, and maltose also showed varied potency in inducing *CAMP* gene expression [76]. Glucose supplementation upregulated *DEFB1* and *CAMP* gene expression in human keratinocytes, leading to greater antimicrobial activity against both *Listeria monocytogenes* and *S*. *aureus* [60]. Interestingly, high glucose induced *DEFB1* expression in human kidney and colon cells [79]. Similarly, *Defb1* was also upregulated in the kidneys of hyperglycemic rats [80]. However, the expression of human *CAMP*, *DEFB3*, and *DEFB4* were downregulated by glucose [179,180]. Multiple sugars, such as lactose, glucose, galactose, trehalose, maltose, sucrose, and fructose, increased HDP gene expression in chicken cells [77]. In pigs, lactose induced several *DEFB* genes in the jejunal mucosa and mitigated the negative effect of Rotavirus on animal growth [78]. Lactose-mediated HDP induction involves the MAPK pathway and histone acetylation. In HT-29 colonic epithelial cells, an inhibition of p38 MAPK and JNK reduced *CAMP* induction by lactose [76], and histone H4 acetylation was increased in chicken HD11 macrophage cells in response to lactose [77]. Additional studies are needed to clarify the mechanisms by which sugars upregulate HDP.

#### Minerals

Zinc exerts a plethora of benefits for mounting an effective immune response to infectious agents [181]. Among them is zinc’s ability to induce HDP synthesis. Zinc was found to induce *CAMP* in human Caco-2 colonic epithelial cells [81] and *DEFB4* in LPS-induced inflammatory skin explants [82] (Table 1 [[15], [16], [17], [18], [19], [20], [21], [22], [23], [24], [25], [26], [27], [28], [29], [30], [31], [32], [33], [34], [35], [36], [37], [38], [39], [40], [41], [42], [43], [44], [45], [46], [47], [48], [49], [50], [51], [52], [53], [54], [55], [56], [57], [58], [59], [60], [61], [62], [63], [64], [65], [66], [67], [68], [69], [70], [71], [72], [73], [74], [75], [76], [77], [78], [79], [80], [81], [82], [83], [84], [85], [86], [87], [88], [89], [90], [91], [92], [93], [94], [95], [96], [97], [98], [99], [100], [101], [102], [103], [104], [105], [106], [107], [108], [109], [110], [111], [112], [113], [114], [115], [116], [117], [118], [119], [120], [121], [122], [123], [124], [125], [126], [127], [128], [129], [130], [131], [132], [133], [134], [135], [136], [137], [138], [139], [140], [141], [142], [143], [144], [145], [146], [147], [148], [149], [150], [151], [152], [153], [154], [155], [156], [157]]). Zinc also upregulated DEFB concentrations in porcine intestinal epithelial cells [65]. On the contrary, zinc deficiency was accompanied by a decrease in Paneth cell DEFA synthesis in both humans [182] and mice [183]. Calcium is capable of inducing multiple *DEFB* gene transcription in human keratinocytes [83,84], while *DEFB4* expression was reduced when calcium was chelated [184]. Chelation of calcium prevented cathelicidin-mediated killing of human Jurkat T leukemia cells [185] and inhibited DEFB-mediated fungicidal activity against *Candida albicans* [186].

#### Bile acids

Several bile acids have been reported to upregulate HDP gene expression. A primary bile acid, chenodeoxycholic acid, and a secondary bile acid, ursodeoxycholic acid, induced *CAMP* transcription in human biliary epithelial cells [86] (Table 1 [[15], [16], [17], [18], [19], [20], [21], [22], [23], [24], [25], [26], [27], [28], [29], [30], [31], [32], [33], [34], [35], [36], [37], [38], [39], [40], [41], [42], [43], [44], [45], [46], [47], [48], [49], [50], [51], [52], [53], [54], [55], [56], [57], [58], [59], [60], [61], [62], [63], [64], [65], [66], [67], [68], [69], [70], [71], [72], [73], [74], [75], [76], [77], [78], [79], [80], [81], [82], [83], [84], [85], [86], [87], [88], [89], [90], [91], [92], [93], [94], [95], [96], [97], [98], [99], [100], [101], [102], [103], [104], [105], [106], [107], [108], [109], [110], [111], [112], [113], [114], [115], [116], [117], [118], [119], [120], [121], [122], [123], [124], [125], [126], [127], [128], [129], [130], [131], [132], [133], [134], [135], [136], [137], [138], [139], [140], [141], [142], [143], [144], [145], [146], [147], [148], [149], [150], [151], [152], [153], [154], [155], [156], [157]]). Another secondary bile acid, lithocholic acid (LCA), upregulated *CAMP* in human HT-29 colonic epithelial cells [16] and primary keratinocytes [85] but not in human Caco-2 colonic epithelial cells [85]. In mouse 3T3-L1 adipocytes, α-tauromuricholic acid and taurohyodeoxycholic acid, increased *Camp* mRNA expression, but cholic acid, deoxycholic acid, and taurodeoxycholic acid had no effect [87]. Two nuclear receptors, farnesoid X receptor and VDR, are involved in the upregulation of *CAMP* by bile acids (Figure 2). Although chenodeoxycholic acid induced *CAMP* through farnesoid X receptor, ursodeoxycholic acid activated VDR [187]. LCA likewise signaled through VDR to improve *CAMP* gene expression [16,85]. Moreover, MEK-ERK signaling was involved in *CAMP* gene regulation by LCA in primary human keratinocytes [85], while VDR recruited PU.1 to the *CAMP* gene promoter in HT-29 colon epithelial cells [16].

#### Phytochemicals

Polyphenols are a diverse group of naturally occurring compounds found in plants with anti-inflammatory, antioxidant, and anticancer properties [188]. Several classes of polyphenols, such as flavonoids, stilbenes, and phenolic acids, are capable of inducing HDP synthesis [189] (Table 1). For example, epigallocatechin gallate (EGCG), a flavonoid found in green tea, enhanced DEFB synthesis in humans [88,89], pigs [90], and chickens [91]. Quercetin is another flavonoid with an ability to improve HDP expression in rats [92], chickens [91,93], and zebrafish [94]. Saponarin (a flavone), genistein (an isoflavone), and apigenin (a trihydroxyflavone) enhanced *CAMP* transcription in human keratinocytes [[95], [96], [97]]. Additionally, xanthohumol (a prenylated flavonoid), calycosin (an isoflavone), datiscetin (a tetrahydroxyflavone), and isoliquiritigenin (a chalcone flavonoid) have been found to induce multiple DEFB genes in pigs [98], chickens [99], and humans [95,100], respectively. Resveratrol, a stilbene in grapes, stimulated HDP gene expression in humans, mice, rats, and chickens [91,92,[101], [102], [103], [104]]. Similarly, polydatin and pterostilbene, 2 resveratrol derivatives, were shown to increase HDP production in human keratinocytes [105] and monocytes [103] as well as in porcine macrophages [98]. Isorhapontigenin, a stilbenoid, augmented the expression concentrations of several DEFBs in porcine intestinal epithelial cells [98]. Phenolic acids, such as ellagic acid, caffeic acid, and anacardic acid, increased the DEFB expression in humans [106], mice [107], and chickens [91]. Curcumin, belonging to the curcuminoid group of polyphenols, stimulated *CAMP* expression in human monocytes, colonic epithelial cells, and keratinocytes [108] and enhanced *HAMP*, *LEAP2*, and DEFB mRNA concentrations in grass carp [109].

A few nonpolyphenol phytochemicals have also been found to upregulate HDP synthesis. For instance, forskolin (FSK), a diterpenoid produced by the Indian Coleus plant, was shown to enhance the expression of *CAMP* in human HT-29 epithelial cells [110], but suppress *CAMP* and *DEFB1* concentrations in butyrate-differentiated HT-29 cells [161]. Chicken macrophage cells treated with FSK increased avian β-defensin 9 (*AvBD9*) transcription in chicken macrophage cells as well as in the crop of orally supplemented chickens [26]. Two diterpenoids, andrographolide and oridonin, were found to induce *DEFB3* expression in human colonic epithelial cells [100]. The supernatant of cells treated with andrographolide and isoliquiritigenin limited the growth of 4 pathogenic bacteria [100]. A number of natural products and phytochemicals in particular were recently identified with the HDP-inducing activity in chicken and porcine cells through high throughput screening [98,99]. For instance, 1 plant alkaloids, tetrandrine and sanguinarine, were each capable of inducing chicken HDP gene expression [99], while deoxyshikonin, a plant naphthoquinone, increased PBD expression [98].

Many phytochemicals upregulate HDP synthesis through MAPK signaling pathways. For example, EGCG induced *DEFB1, DEFB3*, and *DEFB4* via MAPK p38/ERK/JNK pathways in human bronchial or gingival epithelial cells [88,89] and required p38 activation for *PBD2* induction in porcine intestinal epithelium [90]. Andrographolide, oridonin, and isoliquiritigenin activated MAPK pathways downstream of the epidermal growth factor (EGF) receptor and recruited c-Fos, c-Jun, and Elk1 or cMyc to the *DEFB3* promoter in human colon epithelial cells [100]. Although andrographolide upregulated *DEFB3* by EGFR/ERK/JNK, oridonin and isoliquiritigenin instead worked through an EGFR/ERK/p38 signaling pathway [100]. Sulforaphane required VDR and ERK1/2 but not p38 to increase *DEFB4* transcription in human intestinal epithelium [17]. In chicken macrophage cells, inhibitors of p38 or JNK nearly abolished FSK-induced *AvBD9* [26]. MAP kinases are required for the upregulation of HDP by many phytochemicals, albeit the specific signaling cascade varies by treatment.

A NF-κB pathway was activated by resveratrol, genistein, or sulforaphane for HDP upregulation. Resveratrol-induced *CAMP* in human keratinocytes required the spingosine-1-phosphate (S1P) signaling, followed by transactivation of NF-κB and transcription factor C/EPBα [102]. A similar ER-β→S1P→NF-κB →C/EPBα mechanism was found for *CAMP* induction in keratinocytes treated with genistein [97]. Sulforaphane incubated with a NF-κB inhibitor reduced *DEFB4* transcription in human intestinal epithelium [17]. NF-κB was also required for quercetin-induced *AvBD9* induction in chicken monocytes [91]. However, dietary quercetin supplementation downregulated NF-κB mRNA expression in the chicken ileum although multiple AvBD genes were enhanced [93]. The involvement of NF-κB in HDP regulation by phytochemicals should be confirmed in vivo for additional species.

Interestingly, resveratrol, EGCG, quercetin, anacardic acid, and garcinol are natural cyclooxygenase (COX)-2 inhibitors that are able to upregulate HDPs in chicken monocytes [91]. Soponarin and apigenin were also found to inhibit COX-2 in human keratinocytes stimulated with inflammatory cytokines [95], mouse macrophages, and rat basophils [96]. Although inhibition of COX-2 signaling has previously been linked to HDP expression [190], its involvement in HDP regulation after treatment with these polyphenols remains to be studied.

Nuclear factor erythroid 2-related factor 2 (Nrf2) facilitates HDP expression in the liver in response to phytochemical treatment. Quercetin treatment increased binding of Nrf2 to an antioxidant response element at the *HAMP* promoter in parallel to enhanced *HAMP* transcription in human hepatocytes [92]. Curcumin likewise upregulated Nrf2 mRNA concentrations while increasing *HAMP*, *LEAP2*, and DEFB in grass carp liver [109].

FSK elevates intracellular cAMP concentrations, and binding of CREB to the human cathelicidin promoter promoted active gene transcription [110]. However, cAMP inhibited by 2',3'-dideoxyadenosine improved quercetin-induced *AvBD9* in chicken monocytes [91]. The role of cAMP in HDP regulation needs to be clarified for different species and HDP genes.

#### Epigenetic modulators

Epigenetic modifications, such as acetylation of histones and methylation of histones and DNA, play an essential role in regulating chromatin accessibility and gene expression [191]. Histone acetylation is facilitated by histone acetyltransferases to favor a more relaxed chromatin structure, whereas the removal of acetyl groups by HDACs favors a more condensed chromatin configuration [191]. Histones and DNA can also be methylated by DNA methyltransferases (DNMT) and histone methyltransferases (HMT), respectively, to affect gene transcription [191]. HDAC inhibitors have been demonstrated to be among the most potent inducers of HDPs in several high throughput screenings [99,114,121,124,126,192]. Members of all 4 major classes of HDAC inhibitors, including benzamides, hydroxamates, cyclic peptides, and SCFAs, have been shown to be HDP inducers. For example, trichostatin A, a hydroxamate HDCA inhibitor, induced mRNA expression of HDPs in humans [15,18], pigs [114], chickens [99], and cattle [115] (Table 1). Other hydroxamte HDAC inhibitors, such as vorinostat also known as suberoylanilide hydroxamic acid, induced the mRNA expression of *DEFA1*, *DEFA5*, *DEFA6*, and *DEFB4* in human monocytic cells infected with *Leishmania donovani*, contributing to increased antiparasitic activity [117]. Vorinostat also enhanced the expression of several HDP genes in human intestinal epithelium [112,118] and hepatocytes [119], chicken macrophages, [120,126] and porcine intestinal epithelial cells and lung alveolar macrophages [114].

However, among all HDAC inhibitors, benzamides appear to be the most potent HDP inducers in humans, pigs, and chickens as revealed in several recent high throughput assays [114,121,124,126,192]. For example, entinostat, a benzamide HDAC inhibitor, potently enhanced *DEFB1* and *CAMP* mRNA expression in intestinal epithelial cells [121,122] and protected rabbits from experimental cholera [123]. Its analogous compounds, known as aroylated phenylenediamines, had similar effects in human bronchial epithelial cells, associated with a significant reduction in the intracellular invasion of *Pseudomonas aeruginosa* [125]. Entinostat also similarly induced HDP gene expression in chicken jejunal explants, and an oral inoculation of entinostat led to an increased expression of multiple HDP genes in the crop and jejunum of broiler chicks [124]. Mocetinostat, an entinostat analog, potently increased *AvBD9* mRNA concentrations in chicken jejunal explants [126]. Polyphenols, such as sulforaphane, curcumin, and EGCG, are known natural HDAC inhibitors [193] with an HDP-inducing activity [17,89,108].

In addition to HDAC inhibitors, inhibitors of DNMT and HMT are HDP inducers. For instance, 5-azacytidine, a well-known DNMT inhibitor, increased CAMP and DEFB concentrations in gingival epithelial cells [111], oral carcinoma cells [127,128], keratinocytes [113], and chondrocytes [129]. Additionally, 5-azacytidine stimulated HDP expression in bovine mammary epithelial cells [115], chicken macrophage cells [120], the larvae of silkworm [130], as well as in the caput, cauda, and testis of the male rats [116]. Polyphenols such as EGCG, quercetin, and genistein, known for their DNMT inhibitory properties [194], also possess the ability to induce HDP synthesis as discussed in greater detail in an earlier section. Similarly, inhibitors of HMT, such as BIX01294 and UNC1999, have been shown to promote the expression of multiple HDPs in chicken macrophages [120].

**FIGURE 3:**
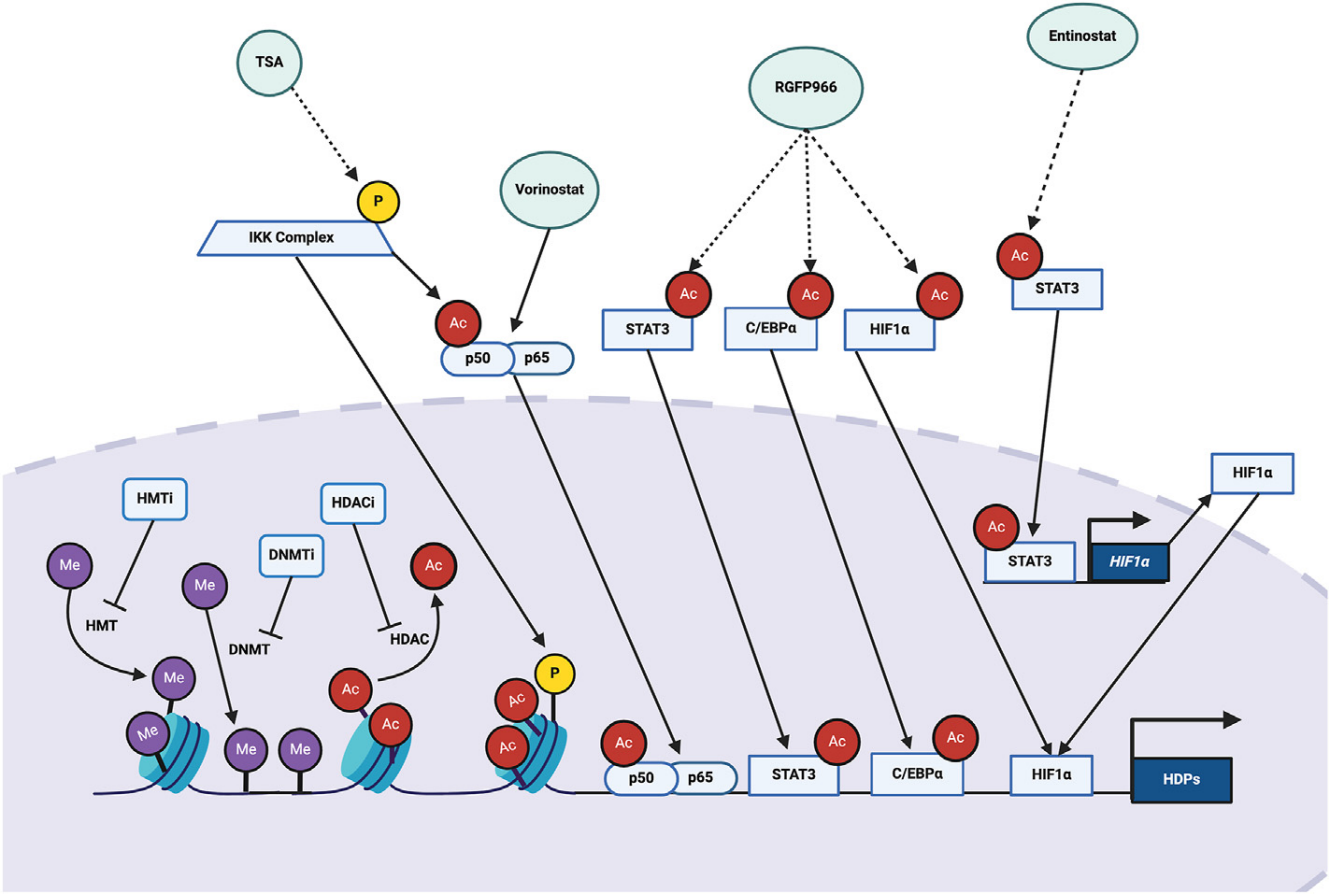
Epigenetic mechanisms of HDP gene induction. See text for details. Ac, acetyl group; C/EBPα, CCAAT enhancer binding protein α; DNMTi, DNA methyltransferase inhibitor; HDACi, histone deacetylase inhibitor; HIF1α, hypoxia-inducible factor-1α; HMTi, histone methyltransferase inhibitor; IKK, Iκ B kinase; P, phosphate group; Me, methyl group; TSA, trichostatin A; p50/p65, NF-κB proteins p50 and p65 heterodimer; STAT, signal transducer and activator of transcription.

Inhibition of HDACs, DNMTs, and HMTs may facilitate chromatin relaxation and promote gene transcription. Several HDAC inhibitors have demonstrated the capacity to promote histone acetylation at HDP promoter regions, accompanied by increased HDP transcription [18,112,117,195]. HDAC inhibition may also activate transcription factors such as NF-κB to turn on the transcription of HDPs such as *DEFB4* owing to the presence of multiple NF-κB binding sites in the promoter region [118] (Figure 3). Consistently, trichostatin A increased activation and translocation of NF-κB through acetylation of p65 and phosphorylation of the IKK complex, ultimately leading to an increased binding of NF-κB to the *DEFB4* promoter in human colonic epithelial cells [112]. Entinostat and its structural analog, HO53, promoted STAT3 activation leading to increased *HIF1α* expression and binding of HIF-1α to the *CAMP* promoter [122,125]. Similarly, RGFP966, an HDAC3 inhibitor, increased acetylation of STAT3, C/EBPα, and HIF-1α in human liver cells, enhancing the affinity of these transcription factors to the *LEAP1* promoter [119].

DNMT inhibitors, such as AZA, reduced DNA methylation at *DEFB1* [113] and *CAMP* [128,129] promoter regions, resulting in an increase in their transcription. Although the mechanism to support HMT inhibitors, BIX01294 and UNC1999, has not been well studied, a strong correlation has been found between histone demethylase, JMJD3, and expression of *DEFB3*, *S100A7*, *S100A8*, and *CAMP* in human keratinocytes [196]. Moreover, *JMJD3* knockdown led to significantly increased histone methylation concentrations and reduced mRNA expression of these HDPs, highlighting the importance of histone methylation in shaping HDP gene expression patterns [196].

#### Probiotics

Probiotics are beneficial microbes that promote intestinal health through a myriad of mechanisms [197]. A variety of lactic acid bacteria have been found to induce HDP expression. For example, several *Lactobacillus* species induced HDP in human intestinal epithelia [[131], [132], [133], [134],^136^], gingival epithelia [135,137], vaginal epithelia [113,138], cervical epithelia [139], and epidermal cells [140] (Table 1). Oral supplementation of *Lactobacillus delbruckii* subsp. *bulgaricus* 8481 decreased *IL-8* and increased *DEFB4* in elderly patients [141]. In infants, *Bifidobacterium animalis* subsp. *lactis* BB-12 elevated both CAMP and DEFB4 protein concentrations [142]. *E. coli* Nissle 1917 were found to enhance *DEFB4* expression in human intestinal epithelial cells [143,198].

Probiotics also induced HDP expression in rodents, pigs, chickens, cattle, and sheep. For instance, lactic acid bacteria were found to enhance HDP production and alleviate colitis [144], vancomycin-resistant enterococcus infection [199], and cirrhosis in mice [145]. In pigs, *Lactobacillus reuteri* I5007, *Lactobacillus amylovorus* SLZX20, and *Lactobacillus plantarum* ZLP001 increased the mRNA expression of multiple DEFBs in porcine intestinal epithelial cell lines [147,149,200], while *L. plantarum* counteracted intestinal barrier dysfunction induced by enterotoxigenic *E. coli* [148]. Treatment of intestinal cells with *Bacillus subtilis* CP9 enhanced *PG1* transcription, but not *PBD3*, while having anti-ETEC properties [150]. Co-administration of *Lactobacillus salivarius* B1 and *B*. *subtilis* RJGP16 significantly induced the expression of *PBD2* in the duodenum of neonatal piglets [151]. Likewise, both *L. reuteri* D8 and *Lactobacillus rhamnosus* Gorbach-Goldin (GG) increased body weight gain and decreased the incidence of diarrhea by upregulating HDPs in the jejunum of piglets [152,201].

In chickens, modulation of intestinal HDPs by *L. reuteri* is gene specific: *AvBD1* and *CATH3* mRNA expressions were increased, while *CATH2* and *AvBD10* were decreased, with no change observed with *AvBD2* or *AvBD12* [153,202]. In bovine mammary epithelial cells, *Lactobacillus casei* BL23 sustained the expression of DEFs during infection with *S. aureus* [154]. Furthermore, *DEFB1* was increased in colon mucosa from dairy cows supplemented with live *Saccharomyces cerevisiae* CNCM I-1077 [155]. *S*. *cerevisiae* similarly upregulated *SBD1* in ovine ruminal epithelial cells [203]. However, not all probiotics have the capacity to induce HDP synthesis. For instance, *Bifidobacterium breve* M16V did not affect the expression of *DEFB1, DEFB4*, or *CAMP* in preterm human infants [204], and 3 of 7 *L. casei* strains tested failed to upregulated *Reg3b* or a *DEFA* in the ileum of mice [146].

**FIGURE 4:**
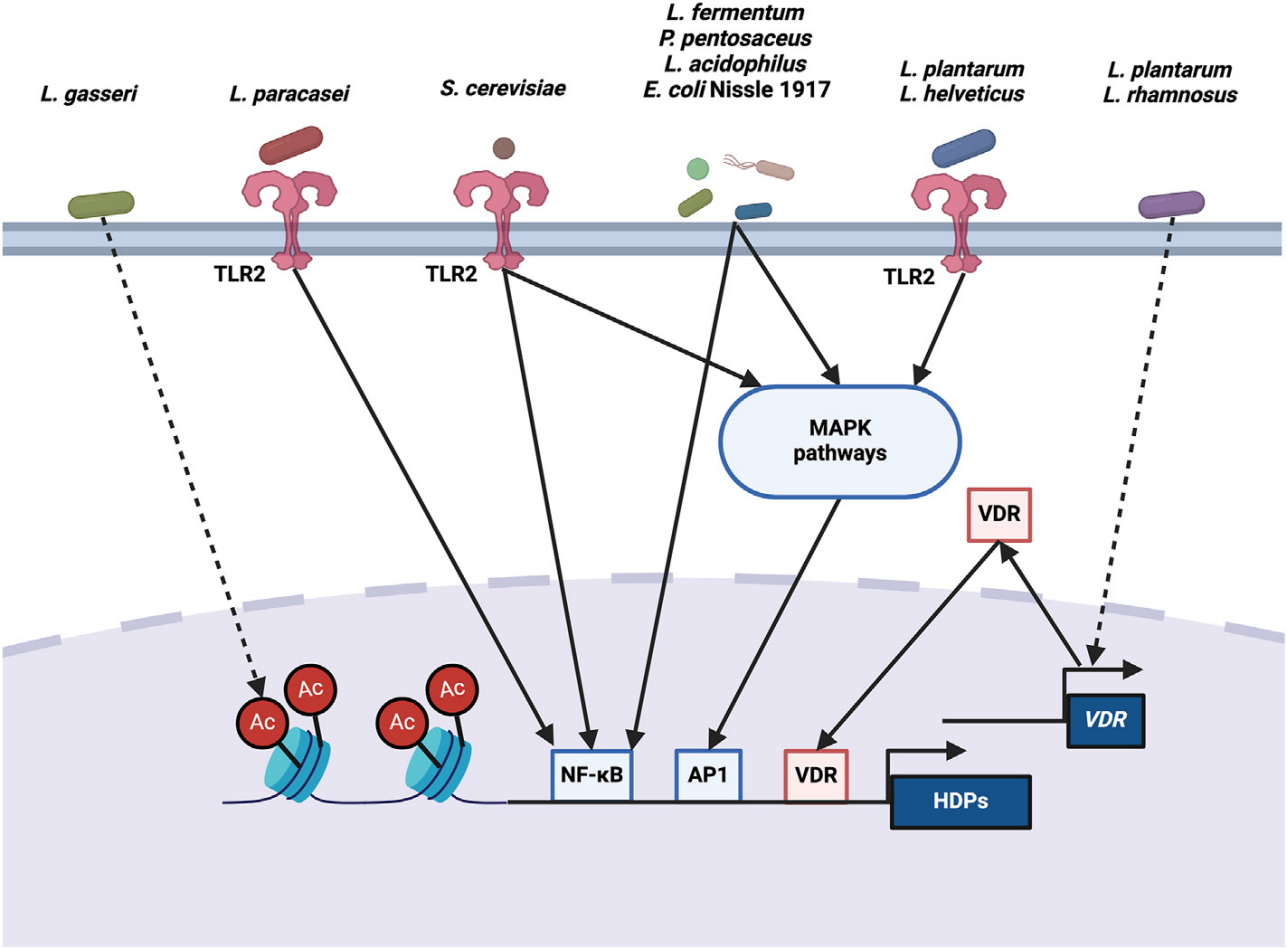
Molecular mechanisms of HDP gene expression by probiotics. See text for details. Ac, acetyl group; AP1, activator protein-1; HDP, host defense peptide; MAPK, mitogen-activated protein kinases; NF-κB, nuclear factor-kappa B; TLR, toll-like receptor, VDR, vitamin D receptor.

Probiotics such as *Lactobacillus* [133,147] or *S. cerevisiae* [203] triggered TLR2 along with MAPK and/or NF-κB signaling pathways to induce HDP gene expression in human, porcine, and ovine cells (Figure 4). *L. rhamnosus* GG and *L. plantarum* increased VDR protein expression in both mouse and human intestinal epithelial cells and induce *CAMP* gene expression through VDR [205]. On the contrary, *L. gasseri,* but not *L. reuteri*, induced *DEFB1* gene expression associated with increased concentrations of histone H3 acetylation, H3K4me3, and H2A.Z in the proximal promoter region of the *DEFB1* gene [113]. *E. coli* Nissle 1917, *Pediococcus pentosaceus*, *Lactobacillus fermentum*, and *Lactobacillus acidophilus* required NF-κB signaling in addition to AP-1 activation to induce *DEFB4* [143,198]. An increase in HDP was paralleled by expression of nucleotide-binding oligomerization domain (NOD1/2) in cells treated with *L. rhamnosus* GG., *B. longum* spp. Infantis S12, or BI 5764 [136,144]. However, *L. reuteri* I5007 enhanced HDPs though upregulation of peroxisome proliferator-activated receptor-γ and GPR41 in porcine epithelial cells [200], while *L. casei* BL23 had no effect on NOD2 nor NF-κB [154]. These discrepancies may be due to differences in probiotic-host interaction or variation in signaling mechanisms between host species. Regardless, the escalating interest in the use of probiotics to promote host health warrants continued research into the mechanisms behind probiotic-induced HDP expression.

#### Prebiotics

Supplementation of inulin to a Western-style diet increased the expression of multiple Paneth cell DEFAs and *DEFB1* as well as tight junction proteins in the intestinal tissues of mice, resulting in improved intestinal barrier integrity and reduced endotoxemia [24] (Table 1). Similarly, long-chain, but not short-chain, inulin-type fructans delayed the onset of type 1 diabetes by promoting gut health, including a significant increase in *Defb1* and *Camp* in the colon of mice [156]. Additionally, oral administration of polysaccharides extracted from *Dendrobium huoshanense,* a medicinal plant, enhanced DEFB production and barrier function of the intestinal tract of mice [157]. The HDP-inducing activity of other prebiotics remains to be studied.

### Synergistic induction of HDP synthesis by different classes of dietary compounds

Several different classes of dietary compounds have been found to synergize with each other to potentiate HDP synthesis (Table 2) [16,26,35,36,76,77,91,99,100,103,105,[120], [121], [122],^206^,^207^]. For example, vitamin D-3 is synergistic with butyrate or PBA in *CAMP* synthesis in bronchial epithelial cells and macrophages, showing a bacteriostatic effect against *M*. *tuberculosis* [36,206]. The combination of vitamin D-3 and PBA was also shown to induce HDPs in human dendritic cells, leading to effective killing of *S*. *aureus* [207]. Vitamin D-3 further synergized with entinostat, resveratrol, or pterostilbene to induce CAMP synthesis in human colon cells, monocytes, or keratinocytes [103,121,122]. Synergy in augmenting *CAMP* expression was also observed when vitamin D-3 was combined with calcium in normal and cystic fibrosis bronchial epithelial cells [208].

**TABLE 2:** Synergistic induction of HDP synthesis by combinations of different classes of dietary compounds

Compounds	Cells or tissues	HDPs	References
Butyrate + lithocholic acid	Human HT-29	*CAMP*	[16]
Butyrate + forskolin	Chicken HD11, HTC, jejunal explants	*AvBD9*	[26]
Butyrate + propionate + acetate	Chicken HD11 macrophages	*AvBD9*	[35]
Butyrate/PBA + lactose	Human HT-29	*CAMP*	[76]
Butyrate + galactose/trehalose/lactose	Chicken HD11 macrophages	*AvBD9, CATHB1*	[77]
Butyrate + COX-2 inhibitors	Chicken HTC, PBMCs	*AvBD9*	[91]
Butyrate + wortmannin/tetrandrine/datiscetin	Chicken HTC, chicken monocytes	*AvBD9*	[99]
Vitamin D-3 + PBA	Human VA10, MDM, PBMCs	*CAMP*	[36,206,207]
Vitamin D-3 + resveratrol/pterostilbene	Human U937, HaCaT	*CAMP*	[103]
Vitamin D-3 + entinostat	Human HT-29	*CAMP*	[121,122]
Vitamin D-3 + calcium	Human NHBE	*CAMP*	[208]
Andrographolide + isoliquiritigenin	Human Caco-2	*DEFB3*	[100]
Polydatin + resveratrol	Human HaCaT	*DEFB4*	[105]
HDACi + HMTi/DNMTi	Chicken HTC, HD11 macrophages	*AvBD9*	[120]

Abbreviations: AvBD, avian β-defensin; CAMP, cathelicidin; CATH, chicken cathelicidin; COX, cyclooxygenase; DEF, defensin; DNMTi, DNA methyltransferase inhibitor; HDACi, histone deacetylase inhibitor; HDP, host defense peptide; HMTi, histone methyltransferase inhibitor; PBA, phenylbutyrate.

In addition to vitamin D-3, butyrate synergizes with several other classes of dietary compounds to enhance HDP synthesis. When combined with lactose, butyrate or 4-PBA showed a synergistic effect on human CAMP expression [76]. Similarly, butyrate synergized with other sugars, such as galactose or trehalose, to upregulate HDP expression in chicken macrophages [77]. Administration of butyrate and FSK showed a synergistic increase in *AvBD9* in the crop and jejunum of chickens [26]. An even higher magnitude of HDP gene induction and protection against necrotic enteritis was observed with the combination of butyrate, lactose, and FSK over any of the 2-compound combinations in chickens [209]. Butyrate in combination with wortmannin, a naturally occurring fungal metabolite and a specific phosphoinositide 3-kinase inhibitor, synergistically increased HDP expression in chickens [99]. Additionally, butyrate synergized with tetrandrine or datiscetin in chicken HDP gene induction [99]. Several polyphenols and COX-2 inhibitors, namely quercetin, resveratrol, anacardic acid, EGCG, and garcinol had a strong synergy with butyrate in HDP transcription in chicken cells [91]. Additionally, butyrate synergized with other SCFAs such as acetate and propionate in enhancing chicken HDP expression both in vitro and in vivo [35].

Furthermore, polydatin, a natural precursor to resveratrol, synergistically increased *DEFB4* production in human keratinocytes when combined with resveratrol [105]. Andrographolide and isoliquiritigenin cooperated to enhance *DEFB3* expression and antibacterial activity of human colonic epithelial cells [100]. Different classes of epigenetic compounds also appear to act in a corporative manner in increasing HDP gene expression. For instance, an HDAC inhibitor paired with an inhibitor of either an HMT or a DNMT led to a drastic synergy in the transcription of multiple HDP genes in chicken macrophages [120]. Additionally, probiotics such as *E. coli* Nissle 1917 synergizes with HDCA inhibitors to enhance *DEFB4* expression in human intestinal cell lines, but not in human colonic biopsies [118].

The mechanism for synergy between HDP inducers remains elusive. HDAC inhibitors including butyrate increase histone acetylation in favor of active gene transcription [30], so a proposed mechanism for synergy with butyrate involved additional enrichment of histone acetylation. Butyrate and lactose treatment led to the hyperacetylation of H4 preceding a synergistic response in chicken HDP gene induction [77]. However, no additional histone acetylation was observed for butyrate and quercetin in relation to their synergy in chicken HDP transcription [91]. Changes in histone acetylation are insufficient to describe the synergy observed between butyrate and a secondary HDP inducer.

Other studies looked at the signaling pathways activated by individual compounds compared with compound combinations. A similar involvement of p38 MAPK, JNK, NF-κB, and cAMP signaling pathways was observed in response to butyrate and lactose as for butyrate alone [77]. Likewise, the signaling pathways activated by lactose and PBA for synergistic induction of *CAMP* coincided with those found for the individual treatments [210].

The mechanism appears to be due to the cooperation between epigenetic modulation and regulation of signaling pathways and/or specific transcription factors as opposed to synergy in boosting any individual pathway. For example, induction of *CAMP* transcription involved cooperation among VDR, C/EBPα, PU.1, and chromatin remodeling [171]. On the contrary, inhibition of ERK1/2 and MAPK-p38 by resveratrol was found to contribute to the synergy with vitamin D-3 in augmenting *CAMP* gene expression [103]. The synergistic effect of andrographolide and isoliquiritigenin on *DEFB3* expression was supported by higher phosphorylation of H3S10 and recruitment of transcription factors Fos and ELK1 than use of either molecule alone [100]. A plausible mechanism for synergy between HDP inducers could be due to increased transcription factor binding when 1 or both modulators can also induce epigenetic changes in favor of an accessible HDP gene promoter.

## Conclusions and Future Prospects

Dietary modulation of endogenous HDP synthesis has potential to be developed as an alternative approach to antimicrobial therapy. Several HDP inducers, such as butyrate, 4-PBA, and vitamin D-3, have demonstrated protective efficacy against infectious diseases across multiple animal species. It is noted that nutritional modulation of endogenous HDP synthesis is unlikely to drive antimicrobial resistance, as these HDP-inducing dietary factors act on the host without exerting direct antimicrobial activity. Additionally, unlike other immune boosters that often nonspecifically trigger inflammation, many of the HDP-inducing compounds stimulate the synthesis of HDPs without eliciting a proinflammatory response while some even have anti-inflammatory effects [31,69,100]. This is beneficial, as inflammation can lead to detrimental effects on the host, such as tissue damage. However, additional human and animal trials are warranted to realize the clinical potential of HDP inducers as novel host-directed antimicrobials.

It is noted that different classes of dietary compounds have a strong capacity to induce HDPs in multiple cell types and animal species, although cell-specific and species-specific HDP induction is evident. Importantly, many compounds show a synergy in promoting HDP synthesis when combined. Mechanisms of dietary compound-mediated HDP induction are being investigated and a detailed understanding of the molecular mechanisms may allow further HDP-inducing efficiency. Current evidence indicates the involvement of histone acetylation as well as MAPK, NF-κB, VDR, cAMP, and COX-2 signaling pathways. Transcription factors such as AP-1, CREB, STAT3, and sphingosine-1-phosphate have also been implicated in nutritional regulation of HDPs. Further investigation may yield dietary compounds or their combinations as promising candidates as effective alternatives to antibiotics for both human and animal applications.

## Author contributions

The authors’ responsibilities were as follows – GZ: conceived the study; MW, IT, AB, GZ: drafted the manuscript; IT, MW: contributed to visualization; GZ: edited the manuscript and has primary responsibility for final content; and all authors: read and approved the final manuscript.

## Conflict of interest

The authors report no conflicts of interest.

## Funding

This research was supported by the USDA National Institute of Food and Agriculture grants (2020-67016-31619 and 2023-67015-39095), the Ralph F. and Leila W. Boulware Endowment Fund, and Oklahoma Agricultural Experiment Station Project H-3112. MW was supported by a USDA National Institute of Food and Agriculture Predoctoral Fellowship grant (2021-67034-35184). However, the funders had no role in the design of the study, the collection or interpretation of data, the writing of the manuscript, or the decision to publish the results.

## References

[1] Schrader S.M., Vaubourgeix J., Nathan C. (2020). Biology of antimicrobial resistance and approaches to combat it. Sci. Transl. Med..

[2] Ghosh C., Sarkar P., Issa R., Haldar J. (2019). Alternatives to conventional antibiotics in the era of antimicrobial resistance. Trends Microbiol.

[3] Christaki E., Marcou M., Tofarides A. (2020). Antimicrobial resistance in bacteria: mechanisms, evolution, and persistence. J. Mol. Evol..

[4] Darby E.M., Trampari E., Siasat P., Gaya M.S., Alav I., Webber M.A. (2023). Molecular mechanisms of antibiotic resistance revisited. Nat. Rev. Microbiol..

[5] Watkins R.R., Bonomo R.A. (2020). Overview: the ongoing threat of antimicrobial resistance. Infect. Dis. Clin. North. Am..

[6] Vidovic N., Vidovic S. (2020). Antimicrobial resistance and food animals: influence of livestock environment on the emergence and dissemination of antimicrobial resistance. Antibiotics (Basel).

[7] McEwen S.A., Collignon P.J. (2018). Antimicrobial resistance: a one health perspective. Microbiol. Spectr..

[8] Bergman P., Raqib R., Rekha R.S., Agerberth B., Gudmundsson G.H. (2020). Host directed therapy against infection by boosting innate immunity. Front. Immunol..

[9] Wu J., Ma N., Johnston L.J., Ma X. (2020). Dietary nutrients mediate intestinal host defense peptide expression. Adv. Nutr..

[10] Magana M., Pushpanathan M., Santos A.L., Leanse L., Fernandez M., Ioannidis A. (2020). The value of antimicrobial peptides in the age of resistance. Lancet Infect. Dis..

[11] Lazzaro B.P., Zasloff M., Rolff J. (2020). Antimicrobial peptides: application informed by evolution. Science.

[12] Mookherjee N., Anderson M.A., Haagsman H.P., Davidson D.J. (2020). Antimicrobial host defence peptides: functions and clinical potential. Nat. Rev. Drug Discov..

[13] Robinson K., Ma X., Liu Y., Qiao S., Hou Y., Zhang G. (2018). Dietary modulation of endogenous host defense peptide synthesis as an alternative approach to in-feed antibiotics. Anim. Nutr..

[14] Koh A., De Vadder F., Kovatcheva-Datchary P., Bäckhed F. (2016). From dietary fiber to host physiology: short-chain fatty acids as key bacterial metabolites. Cell.

[15] Schauber J., Svanholm C., Termen S., Iffland K., Menzel T., Scheppach W. (2003). Expression of the cathelicidin LL-37 is modulated by short chain fatty acids in colonocytes: relevance of signalling pathways. Gut.

[16] Termén S., Tollin M., Rodriguez E., Sveinsdóttir S.H., Jóhannesson B., Cederlund A. (2008). PU.1 and bacterial metabolites regulate the human gene CAMP encoding antimicrobial peptide LL-37 in colon epithelial cells. Mol. Immunol..

[17] Schwab M., Reynders V., Loitsch S., Steinhilber D., Schröder O., Stein J. (2008). The dietary histone deacetylase inhibitor sulforaphane induces human beta-defensin-2 in intestinal epithelial cells. Immunology.

[18] Kallsen K., Andresen E., Heine H. (2012). Histone deacetylase (HDAC) 1 controls the expression of beta defensin 1 in human lung epithelial cells. PLoS One.

[19] Kida Y., Shimizu T., Kuwano K. (2006). Sodium butyrate up-regulates cathelicidin gene expression via activator protein-1 and histone acetylation at the promoter region in a human lung epithelial cell line, EBC-1. Mol. Immunol..

[20] Jiang W., Sunkara L.T., Zeng X., Deng Z., Myers S.M., Zhang G. (2013). Differential regulation of human cathelicidin LL-37 by free fatty acids and their analogs. Peptides.

[21] Zhang K., Hussain T., Wang J., Li M., Wang W., Ma X. (2020). Sodium butyrate abrogates the growth and pathogenesis of *Mycobacterium bovis* via regulation of cathelicidin (LL37) expression and NF-κB signaling. Front. Microbiol..

[22] Zhou Z., Yang H., Li H., Li X., Li X., Wu B. (2019). Sodium butyrate ameliorates Corynebacterium pseudotuberculosis infection in RAW264.7 macrophages and C57BL/6 mice. Microb. Pathog..

[23] Takakuwa A., Nakamura K., Kikuchi M., Sugimoto R., Ohira S., Yokoi Y. (2019). Butyric acid and leucine induce α-defensin secretion from small intestinal Paneth cells. Nutrients.

[24] Beisner J., Filipe Rosa L., Kaden-Volynets V., Stolzer I., Günther C., Bischoff S.C. (2021). Prebiotic inulin and sodium butyrate attenuate obesity-induced intestinal barrier dysfunction by induction of antimicrobial peptides. Front. Immunol..

[25] Sunkara L.T., Achanta M., Schreiber N.B., Bommineni Y.R., Dai G., Jiang W. (2011). Butyrate enhances disease resistance of chickens by inducing antimicrobial host defense peptide gene expression. PLoS One.

[26] Sunkara L.T., Zeng X., Curtis A.R., Zhang G. (2014). Cyclic AMP synergizes with butyrate in promoting beta-defensin 9 expression in chickens. Mol. Immunol..

[27] Alva-Murillo N., Medina-Estrada I., Báez-Magaña M., Ochoa-Zarzosa A., López-Meza J.E. (2015). The activation of the TLR2/p38 pathway by sodium butyrate in bovine mammary epithelial cells is involved in the reduction of *Staphylococcus aureus* internalization. Mol. Immunol..

[28] Dai H., Wei G., Wang Y., Ma N., Chang G., Shen X. (2020). Sodium butyrate promotes lipopolysaccharide-induced innate immune responses by enhancing mitogen-activated protein kinase activation and histone acetylation in bovine mammary epithelial cells. J. Dairy Sci..

[29] Zeng X., Sunkara L.T., Jiang W., Bible M., Carter S., Ma X. (2013). Induction of porcine host defense peptide gene expression by short-chain fatty acids and their analogs. PLoS One.

[30] Xiong H., Guo B., Gan Z., Song D., Lu Z., Yi H. (2016). Butyrate upregulates endogenous host defense peptides to enhance disease resistance in piglets via histone deacetylase inhibition. Sci. Rep..

[31] Dou X., Han J., Song W., Dong N., Xu X., Zhang W. (2017). Sodium butyrate improves porcine host defense peptide expression and relieves the inflammatory response upon Toll-like receptor 2 activation and histone deacetylase inhibition in porcine kidney cells. Oncotarget.

[32] Dou X., Gao N., Lan J., Han J., Yang Y., Shan A. (2020). TLR2/EGFR are two sensors for pBD3 and pEP2C induction by sodium butyrate independent of HDAC inhibition. J. Agric. Food Chem..

[33] Wang S., Zhang C., Yang J., Wang X., Wu K., Zhang B. (2020). Sodium butyrate protects the intestinal barrier by modulating intestinal host defense peptide expression and gut microbiota after a challenge with deoxynivalenol in weaned piglets. J. Agric. Food Chem..

[34] Nakatsuji T., Kao M.C., Zhang L., Zouboulis C.C., Gallo R.L., Huang C.M. (2010). Sebum free fatty acids enhance the innate immune defense of human sebocytes by upregulating beta-defensin-2 expression. J. Invest. Dermatol..

[35] Sunkara L.T., Jiang W., Zhang G. (2012). Modulation of antimicrobial host defense peptide gene expression by free fatty acids. PLoS One.

[36] Steinmann J., Halldorsson S., Agerberth B., Gudmundsson G.H. (2009). Phenylbutyrate induces antimicrobial peptide expression, Antimicrob. Agents Chemother..

[37] Sarker P., Ahmed S., Tiash S., Rekha R.S., Stromberg R., Andersson J. (2011). Phenylbutyrate counteracts Shigella mediated downregulation of cathelicidin in rabbit lung and intestinal epithelia: a potential therapeutic strategy. PLoS One.

[38] Dou X., Han J., Ma Q., Cheng B., Shan A., Gao N. (2018). TLR2/4-mediated NF-κB pathway combined with the histone modification regulates β-defensins and interleukins expression by sodium phenyl butyrate in porcine intestinal epithelial cells, Food Nutr. Res.

[39] Pineda Molina C., Hussey G.S., Eriksson J., Shulock M.A., Cárdenas Bonilla L.L., Giglio R.M. (2019). 4-Hydroxybutyrate promotes endogenous antimicrobial peptide expression in macrophages. Tissue Eng. Part A..

[40] Wang J., Huang N., Xiong J., Wei H., Jiang S., Peng J. (2018). Caprylic acid and nonanoic acid upregulate endogenous host defense peptides to enhance intestinal epithelial immunological barrier function via histone deacetylase inhibition. Int. Immunopharmacol..

[41] Alva-Murillo N., Ochoa-Zarzosa A., López-Meza J.E. (2012). Short chain fatty acids (propionic and hexanoic) decrease Staphylococcus aureus internalization into bovine mammary epithelial cells and modulate antimicrobial peptide expression. Vet. Microbiol..

[42] Wang T.T., Nestel F.P., Bourdeau V., Nagai Y., Wang Q., Liao J. (2004). Cutting edge: 1,25-dihydroxyvitamin D3 is a direct inducer of antimicrobial peptide gene expression. J. Immunol..

[43] Gombart A.F., Borregaard N., Koeffler H.P. (2005). Human cathelicidin antimicrobial peptide (CAMP) gene is a direct target of the vitamin D receptor and is strongly up-regulated in myeloid cells by 1,25-dihydroxyvitamin D3. FASEB J.

[44] Liao S., Huang Y., Zhang J., Xiong Q., Chi M., Yang L. (2023). Vitamin D promotes epithelial tissue repair and host defense responses against influenza H1N1 virus and Staphylococcus aureus infections. Respir. Res..

[45] Schauber J., Oda Y., Büchau A.S., Yun Q.C., Steinmeyer A., Zügel U. (2008). Histone acetylation in keratinocytes enables control of the expression of cathelicidin and CD14 by 1,25-dihydroxyvitamin D3. J. Invest. Dermatol..

[46] Martineau A.R., Wilkinson K.A., Newton S.M., Floto R.A., Norman A.W., Skolimowska K. (2007). IFN-gamma- and TNF-independent vitamin D-inducible human suppression of mycobacteria: the role of cathelicidin LL-37. J. Immunol..

[47] Karlsson J., Carlsson G., Larne O., Andersson M., Pütsep K. (2008). Vitamin D3 induces pro-LL-37 expression in myeloid precursors from patients with severe congenital neutropenia. J. Leukoc. Biol..

[48] Filipe Rosa L., Petersen P.P., Görtz L.F., Stolzer I., Kaden-Volynets V., Günther C. (2023). Vitamin A- and D-deficient diets disrupt intestinal antimicrobial peptide defense involving Wnt and STAT5 signaling pathways in mice. Nutrients.

[49] Zhang L., Lu L., Li S., Zhang G., Ouyang L., Robinson K. (2016). 1,25-Dihydroxyvitamin-D3 induces avian beta-defensin gene expression in chickens. PLoS One.

[50] Téllez-Pérez A.D., Alva-Murillo N., Ochoa-Zarzosa A., López-Meza J.E. (2012). Cholecalciferol (vitamin D) differentially regulates antimicrobial peptide expression in bovine mammary epithelial cells: implications during *Staphylococcus aureus* internalization. Vet. Microbiol..

[51] Flores-Villalva S., Remot A., Carreras F., Winter N., Gordon S.V., Meade K.G. (2023). Vitamin D induced microbicidal activity against *Mycobacterium bovis* BCG is dependent on the synergistic activity of bovine peripheral blood cell populations. Vet. Immunol. Immunopathol..

[52] Heilborn J.D., Weber G., Grönberg A., Dieterich C., Ståhle M. (2010). Topical treatment with the vitamin D analogue calcipotriol enhances the upregulation of the antimicrobial protein hCAP18/LL-37 during wounding in human skin in vivo. Exp. Dermatol..

[53] Jacobo-Delgado Y.M., Torres-Juarez F., Rodríguez-Carlos A., Santos-Mena A., Enciso-Moreno J.E., Rivas-Santiago C. (2021). Retinoic acid induces antimicrobial peptides and cytokines leading to *Mycobacterium tuberculosis* elimination in airway epithelial cells. Peptides.

[54] Elloumi H.Z., Holland S.M. (2008). Complex regulation of human cathelicidin gene expression: novel splice variants and 5'UTR negative regulatory element. Mol. Immunol..

[55] Liggins M.C., Li F., Zhang L.J., Dokoshi T., Gallo R.L. (2019). Retinoids enhance the expression of cathelicidin antimicrobial peptide during reactive dermal adipogenesis. J. Immunol..

[56] Lee S.E., Lee J.S., Kim M.R., Kim M.Y., Kim S.C. (2010). Topical retinoids induce beta-defensin 3 expression in mouse skin. Int. J. Dermatol..

[57] Kyme P., Thoennissen N.H., Tseng C.W., Thoennissen G.B., Wolf A.J., Shimada K. (2012). C/EBPepsilon mediates nicotinamide-enhanced clearance of *Staphylococcus aureus* in mice. J. Clin. Invest..

[58] Chen Y., Li P., Zhen R., Wang L., Feng J., Xie Y. (2022). Effects of niacin on intestinal epithelial barrier, intestinal immunity, and microbial community in weaned piglets challenged by PDCoV. Int. Immunopharmacol..

[59] Zhen R., Feng J., He D., Chen Y., Chen T., Cai W. (2022). Effects of niacin on resistance to enterotoxigenic *Escherichia coli* infection in weaned piglets. Front. Nutr..

[60] Cruz Díaz L.A., Flores Miramontes M.G., Chávez Hurtado P., Allen K., Gonzalez Ávila M., Prado Montes de Oca E. (2015). Ascorbic acid, ultraviolet C rays, and glucose but not hyperthermia are elicitors of human beta-defensin 1 mRNA in normal keratinocytes. BioMed Res. Int..

[61] Luo K., Li X., Wang L., Rao W., Wu Y., Liu Y. (2021). Ascorbic acid regulates the immunity, anti-oxidation and apoptosis in abalone *Haliotis discus* hannai Ino. Antioxidants (Basel).

[62] Sherman H., Chapnik N., Froy O. (2006). Albumin and amino acids upregulate the expression of human beta-defensin 1. Mol. Immunol..

[63] Rivas-Santiago C.E., Rivas-Santiago B., León D.A., Castañeda-Delgado J., Hernández Pando R. (2011). Induction of beta-defensins by l-isoleucine as novel immunotherapy in experimental murine tuberculosis. Clin. Exp. Immunol..

[64] Fehlbaum P., Rao M., Zasloff M., Anderson G.M. (2000). An essential amino acid induces epithelial beta-defensin expression. Proc. Natl. Acad. Sci. U.S.A..

[65] Mao X., Qi S., Yu B., He J., Yu J., Chen D. (2013). Zn(2+) and L-isoleucine induce the expressions of porcine beta-defensins in IPEC-J2 cells. Mol. Biol. Rep..

[66] Ren M., Zhang S., Liu X., Li S., Mao X., Zeng X. (2016). Different lipopolysaccharide branched-chain amino acids modulate porcine intestinal endogenous β-defensin expression through the Sirt1/ERK/90RSK pathway. J. Agric. Food Chem..

[67] Zhao Y., Yan M.Y., Jiang Q., Yin L., Zhou X.Q., Feng L. (2021). Isoleucine improved growth performance, and intestinal immunological and physical barrier function of hybrid catfish *Pelteobagrus vachelli* × *Leiocassis longirostris*. Fish Shellfish Immunol.

[68] Zhao J., Zhao Y., Liu H., Cao Q., Feng L., Zhang Z. (2023). Dietary leucine improves fish intestinal barrier function by increasing humoral immunity, antioxidant capacity, and tight junction. Int. J. Mol. Sci..

[69] Gao N., Yang Y., Liu S., Fang C., Dou X., Zhang L. (2022). Gut-derived metabolites from dietary tryptophan supplementation quench intestinal inflammation through the AMPK-SIRT1-autophagy pathway. J. Agric. Food Chem..

[70] Gao N., Dou X., Yin T., Yang Y., Yan D., Ma Z. (2021). Tryptophan promotes intestinal immune defense through calcium-sensing receptor (CaSR)-dependent metabolic pathways. J. Agric. Food Chem..

[71] Rao Z., Li J., Shi B., Zeng Y., Liu Y., Sun Z. (2021). Dietary tryptophan levels impact growth performance and intestinal microbial ecology in weaned piglets via tryptophan metabolites and intestinal antimicrobial peptides. Animals. (Basel).

[72] Wang C., Yang Y., Gao N., Lan J., Dou X., Li J. (2021). L-Threonine upregulates the expression of β-defensins by activating the NF-κB signaling pathway and suppressing SIRT1 expression in porcine intestinal epithelial cells. Food Funct.

[73] Dong Y.W., Jiang W.D., Wu P., Liu Y., Kuang S.Y., Tang L. (2022). Novel insight into nutritional regulation in enhancement of immune status and mediation of inflammation dynamics integrated study in vivo and in vitro of teleost grass carp (*Ctenopharyngodon idella*): administration of threonine. Front. Immunol..

[74] Liu G., Ren W., Fang J., Hu C.A., Guan G., Al-Dhabi N.A. (2017). L-glutamine and L-arginine protect against enterotoxigenic *Escherichia coli* infection via intestinal innate immunity in mice. Amino Acids.

[75] Wang X., Pierre J.F., Heneghan A.F., Busch R.A., Kudsk K.A. (2015). Glutamine improves innate immunity and prevents bacterial enteroinvasion during parenteral nutrition. JPEN J. Parenter. Enteral. Nutr..

[76] Cederlund A., Kai-Larsen Y., Printz G., Yoshio H., Alvelius G., Lagercrantz H. (2013). Lactose in human breast milk an inducer of innate immunity with implications for a role in intestinal homeostasis. PLoS One.

[77] Yang Q., Fong L.A., Lyu W., Sunkara L.T., Xiao K., Zhang G. (2021). Synergistic induction of chicken antimicrobial host defense peptide gene expression by butyrate and sugars. Front. Microbiol..

[78] Yu W., Xiao X., Chen D., Yu B., He J., Zheng P. (2022). Effect of dietary lactose supplementation on growth performance and intestinal epithelium functions in weaned pigs challenged by Rotavirus. Animals.

[79] Barnea M., Madar Z., Froy O. (2008). Glucose and insulin are needed for optimal defensin expression in human cell lines. Biochem. Biophys. Res. Commun..

[80] Page R.A., Malik A.N. (2003). Elevated levels of beta defensin-1 mRNA in diabetic kidneys of GK rats. Biochem. Biophys. Res. Commun..

[81] Talukder P., Satho T., Irie K., Sharmin T., Hamady D., Nakashima Y. (2011). Trace metal zinc stimulates secretion of antimicrobial peptide LL-37 from Caco-2 cells through ERK and p38 MAP kinase. Int. Immunopharmacol..

[82] Poiraud C., Quereux G., Knol A.C., Zuliani T., Allix R., Khammari A. (2012). Human beta-defensin-2 and psoriasin, two new innate immunity targets of zinc gluconate. Eur. J. Dermatol..

[83] Harder J., Meyer-Hoffert U., Wehkamp K., Schwichtenberg L., Schröder J.M. (2004). Differential gene induction of human beta-defensins (hBD-1, -2, -3, and -4) in keratinocytes is inhibited by retinoic acid. J. Invest. Dermatol..

[84] Abiko Y., Nishimura M., Kusano K., Yamazaki M., Arakawa T., Takuma T. (2003). Upregulated expression of human beta defensin-1 and -3 mRNA during differentiation of keratinocyte immortalized cell lines, HaCaT and PHK16-0b. J. Dermatol. Sci..

[85] Peric M., Koglin S., Dombrowski Y., Gross K., Bradac E., Ruzicka T. (2009). VDR and MEK-ERK dependent induction of the antimicrobial peptide cathelicidin in keratinocytes by lithocholic acid. Mol. Immunol..

[86] D’Aldebert E., Biyeyeme Bi Mve M.J., Mergey M., Wendum D., Firrincieli D., Coilly A. (2009). Bile salts control the antimicrobial peptide cathelicidin through nuclear receptors in the human biliary epithelium. Gastroenterology.

[87] Hochberg A., Patz M., Karrasch T., Schäffler A., Schmid A. (2021). Serum levels and adipose tissue gene expression of cathelicidin antimicrobial peptide (CAMP) in obesity and during weight loss, Horm. Metab. Res..

[88] Lombardo T.B., Feghali K., Zhao L., Palomari Spolidorio D.M., Grenier D. (2014). Green tea extract and its major constituent, epigallocatechin-3-gallate, induce epithelial beta-defensin secretion and prevent beta-defensin degradation by Porphyromonas gingivalis. J. Periodontal. Res..

[89] Mou Q., Jiang Y., Zhu L., Zhu Z., Ren T. (2020). EGCG induces β-defensin 3 against influenza A virus H1N1 by the MAPK signaling pathway. Exp. Ther. Med..

[90] Wan M.L., Ling K.H., Wang M.F., El-Nezami H. (2016). Green tea polyphenol epigallocatechin-3-gallate improves epithelial barrier function by inducing the production of antimicrobial peptide pBD-1 and pBD-2 in monolayers of porcine intestinal epithelial IPEC-J2 cells. Mol. Nutr. Food Res..

[91] Yang Q., Burkardt A.C., Sunkara L.T., Xiao K., Zhang G. (2022). Natural cyclooxygenase-2 inhibitors synergize with butyrate to augment chicken host defense peptide gene expression. Front. Immunol..

[92] Bayele H.K., Balesaria S., Srai S.K. (2015). Phytoestrogens modulate hepcidin expression by Nrf2: implications for dietary control of iron absorption. Free Radic. Biol. Med..

[93] Ying L., Wu H., Zhou S., Lu H., Ding M., Wang B. (2022). Toll-like receptors signaling pathway of quercetin regulating avian beta-defensin in the ileum of broilers. Front. Cell Dev. Biol..

[94] Wang J., Zhang C., Zhang J., Xie J., Yang L., Xing Y. (2020). The effects of quercetin on immunity, antioxidant indices, and disease resistance in zebrafish (*Danio rerio*). Fish Physiol. Biochem..

[95] Min S.Y., Park C.H., Yu H.W., Park Y.J. (2021). Anti-inflammatory and anti-allergic effects of saponarin and its impact on signaling pathways of RAW 264.7, RBL-2H3, and HaCaT cells. Int. J. Mol. Sci..

[96] Park C.H., Min S.Y., Yu H.W., Kim K., Kim S., Lee H.J. (2020). Effects of apigenin on RBL-2H3, RAW264.7, and HaCaT cells: anti-allergic, anti-inflammatory, and skin-protective activities. Int. J. Mol. Sci..

[97] Park K., Kim Y.I., Shin K.O., Seo H.S., Kim J.Y., Mann T. (2014). The dietary ingredient, genistein, stimulates cathelicidin antimicrobial peptide expression through a novel S1P-dependent mechanism. J. Nutr. Biochem..

[98] Wang J., Lyu W., Zhang W., Chen Y., Luo F., Wang Y. (2021). Discovery of natural products capable of inducing porcine host defense peptide gene expression using cell-based high throughput screening. J. Anim. Sci. Biotechnol..

[99] Lyu W., Deng Z., Sunkara L.T., Becker S., Robinson K., Matts R. (2018). High throughput screening for natural host defense peptide-inducing compounds as novel alternatives to antibiotics. Front. Cell Infect. Microbiol..

[100] Sechet E., Telford E., Bonamy C., Sansonetti P.J., Sperandio B. (2018). Natural molecules induce and synergize to boost expression of the human antimicrobial peptide beta-defensin-3. Proc. Natl. Acad. Sci. U.S.A..

[101] Lee S.I., Park K.H., Kim S.J., Kang Y.G., Lee Y.M. (2012). Mechanical stress-activated immune response genes via sirtuin 1 expression in human periodontal ligament cells. Clin. Exp. Immunol..

[102] Park K., Elias P.M., Hupe M., Borkowski A.W., Gallo R.L., Shin K.O. (2013). Resveratrol stimulates sphingosine-1-phosphate signaling of cathelicidin production. J Invest Dermatol.

[103] Guo C., Sinnott B., Niu B., Lowry M.B., Fantacone M.L., Gombart A.F. (2014). Synergistic induction of human cathelicidin antimicrobial peptide gene expression by vitamin D and stilbenoids. Mol. Nutr. Food Res..

[104] Zhuang Y., Huang H., Liu S., Liu F., Tu Q., Yin Y., He S. (2021). Resveratrol improves growth performance, intestinal morphology, and microbiota composition and metabolism in mice. Front. Microbiol..

[105] Ravagnan G., De Filippis A., Cartenì M., De Maria S., Cozza V., Petrazzuolo M. (2013). Polydatin, a natural precursor of resveratrol, induces beta-defensin production and reduces inflammatory response. Inflammation.

[106] Promsong A., Chung W.O., Satthakarn S., Nittayananta W. (2015). Ellagic acid modulates the expression of oral innate immune mediators: potential role in mucosal protection. J. Oral Pathol. Med..

[107] de Barros P.P., Rossoni R.D., Garcia M.T., Kaminski V.L., Loures F.V., Fuchs B.B. (2021). The anti-biofilm efficacy of caffeic acid phenethyl ester (CAPE) in vitro and a murine model of oral candidiasis. Front. Cell. Infect. Microbiol..

[108] Guo C., Rosoha E., Lowry M.B., Borregaard N., Gombart A.F. (2013). Curcumin induces human cathelicidin antimicrobial peptide gene expression through a vitamin D receptor-independent pathway. J. Nutr. Biochem..

[109] Ming J., Ye J., Zhang Y., Xu Q., Yang X., Shao X. (2020). Optimal dietary curcumin improved growth performance, and modulated innate immunity, antioxidant capacity and related genes expression of NF-κB and Nrf2 signaling pathways in grass carp (*Ctenopharyngodon idella*) after infection with *Aeromonas hydrophila*. Fish Shellfish Immunol.

[110] Chakraborty K., Maity P.C., Sil A.K., Takeda Y., Das S. (2009). cAMP stringently regulates human cathelicidin antimicrobial peptide expression in the mucosal epithelial cells by activating cAMP-response element-binding protein, AP-1, and inducible cAMP early repressor. J. Biol. Chem..

[111] Yin L., Chung W.O. (2011). Epigenetic regulation of human beta-defensin 2 and CC chemokine ligand 20 expression in gingival epithelial cells in response to oral bacteria. Mucosal Immunol.

[112] Fischer N., Sechet E., Friedman R., Amiot A., Sobhani I., Nigro G. (2016). Histone deacetylase inhibition enhances antimicrobial peptide but not inflammatory cytokine expression upon bacterial challenge. Proc. Natl. Acad. Sci. U.S.A..

[113] Lee J., Jang A., Kim J.W., Han J.H., Chun B.H., Jung H.S. (2017). Distinct histone modifications modulate DEFB1 expression in human vaginal keratinocytes in response to *Lactobacillus* spp. Probiotics Antimicrob. Proteins.

[114] Deng Z., Wang J., Lyu W., Wieneke X., Matts R., Ma X. (2018). Development of a cell-based high-throughput screening assay to identify porcine host defense peptide-inducing compounds. J. Immunol. Res..

[115] Kweh M.F., Merriman K.E., Nelson C.D. (2019). Short communication: inhibition of DNA methyltransferase and histone deacetylase increases beta-defensin expression but not the effects of lipopolysaccharide or 1,25-dihydroxyvitamin D3 in bovine mammary epithelial cells. J. Dairy Sci..

[116] Sangeeta K., Yenugu S. (2019). Male reproductive tract antimicrobial expression in the extremes of ages of rats. Gene.

[117] Roy G., Brar H.K., Muthuswami R., Madhubala R. (2020). Epigenetic regulation of defense genes by histone deacetylase1 in human cell line-derived macrophages promotes intracellular survival of *Leishmania donovani*. PLoS Negl. Trop. Dis..

[118] Stebe-Frick S., Ostaff M.J., Stange E.F., Malek N.P., Wehkamp J. (2018). Histone deacetylase-mediated regulation of the antimicrobial peptide hBD2 differs in intestinal cell lines and cultured tissue. Sci. Rep..

[119] Zhou Y., Wang Q., Yang Q., Tang J., Xu C., Gai D. (2018). Histone deacetylase 3 inhibitor suppresses hepatitis C virus replication by regulating Apo-A1 and LEAP-1 expression. Virol. Sin..

[120] Whitmore M.A., Li H., Lyu W., Khanam S., Zhang G. (2022). Epigenetic regulation of host defense peptide synthesis: synergy between histone deacetylase inhibitors and DNA/histone methyltransferase inhibitors. Front. Immunol..

[121] Ottosson H., Nylén F., Sarker P., Miraglia E., Bergman P., Gudmundsson G.H. (2016). Potent inducers of endogenous antimicrobial peptides for host directed therapy of infections. Sci. Rep..

[122] Miraglia E., Nylén F., Johansson K., Arnér E., Cebula M., Farmand S. (2016). Entinostat up-regulates the CAMP gene encoding LL-37 via activation of STAT3 and HIF-1alpha transcription factors. Sci. Rep..

[123] Sarker P., Banik A., Stromberg R., Gudmundsson G.H., Raqib R., Agerberth B. (2017). Treatment with entinostat heals experimental cholera by affecting physical and chemical barrier functions of intestinal epithelia. Antimicrob. Agents Chemother..

[124] Deng Z., Lyu W., Zhang G. (2022). High-throughput identification of epigenetic compounds to enhance chicken host defense peptide gene expression. Antibiotics. (Basel).

[125] Myszor I.T., Parveen Z., Ottosson H., Bergman P., Agerberth B., Strömberg R. (2019). Novel aroylated phenylenediamine compounds enhance antimicrobial defense and maintain airway epithelial barrier integrity. Sci. Rep..

[126] Lyu W., Mi D., Vinson P.N., Xiao Y., Zhang G. (2022). Large-scale identification of multiple classes of host defense peptide-inducing compounds for antimicrobial therapy. Int. J. Mol. Sci..

[127] Kamino Y., Kurashige Y., Uehara O., Sato J., Nishimura M., Yoshida K. (2014). HBD-2 is downregulated in oral carcinoma cells by DNA hypermethylation, and increased expression of hBD-2 by DNA demethylation and gene transfection inhibits cell proliferation and invasion. Oncol. Rep..

[128] Chen X., Qi G., Qin M., Zou Y., Zhong K., Tang Y. (2017). DNA methylation directly downregulates human cathelicidin antimicrobial peptide gene (CAMP) promoter activity. Oncotarget.

[129] Wang G., Li Y., Yang G., Yang T., He L., Wang Y. (2021). Cathelicidin antimicrobial peptide (CAMP) gene promoter methylation induces chondrocyte apoptosis. Hum. Genomics..

[130] Kausar S., Abbas M.N., Gul I., Liu R., Li Q., Zhao E. (2022). Molecular identification of two DNA methyltransferase genes and their functional characterization in the anti-bacterial immunity of *Antheraea pernyi*. Front. Immunol..

[131] Ghadimi D., Hassan M., Njeru P.N., de Vrese M., Geis A., Shalabi S.I. (2011). Suppression subtractive hybridization identifies bacterial genomic regions that are possibly involved in hBD-2 regulation by enterocytes. Mol. Nutr. Food Res..

[132] Messaoudi S., Madi A., Prévost H., Feuilloley M., Manai M., Dousset X. (2012). In vitro evaluation of the probiotic potential of Lactobacillus salivarius SMXD51. Anaerobe.

[133] Paparo L., Aitoro R., Nocerino R., Fierro C., Bruno C., Canani R.B. (2018). Direct effects of fermented cow’s milk product with *Lactobacillus paracasei* CBA L74 on human enterocytes. Benef. Microbes..

[134] Kobatake E., Kabuki T. (2019). S-layer protein of *Lactobacillus helveticus* SBT2171 promotes human β-defensin 2 expression via TLR2–JNK signaling. Front. Microbiol..

[135] Kobatake E., Kobayashi R., Kabuki T., Kurita-Ochiai T. (2019). *Lactobacillus helveticus* SBT2171 upregulates the expression of β-defensin and ameliorates periodontal disease caused by *Porphyromonas gingivalis*. Microbiol. Immunol..

[136] Huang F.C., Lu Y.T., Liao Y.H. (2020). Beneficial effect of probiotics on *Pseudomonas aeruginosa*-infected intestinal epithelial cells through inflammatory IL-8 and antimicrobial peptide human beta-defensin-2 modulation. Innate Immun.

[137] Pahumunto N., Duangnumsawang Y., Teanpaisan R. (2022). Effects of potential probiotics on the expression of cytokines and human beta-defensins in human gingival epithelial cells and in vivo efficacy in a dog model. Arch. Oral. Biol..

[138] Miquel S., Verlaguet J., Garcin S., Bertran T., Evrard B., Forestier C., Vareille-Delarbre M. (2022). *Lacticaseibacillus rhamnosus* Lcr35 stimulates epithelial vaginal defenses upon *Gardnerella vaginalis* infection. Infect. Immun..

[139] Rizzo A., Losacco A., Carratelli C.R. (2013). *Lactobacillus crispatus* modulates epithelial cell defense against *Candida albicans* through toll-like receptors 2 and 4, interleukin 8 and human beta-defensins 2 and 3. Immunol. Lett..

[140] Rosignoli C., Thibaut de Ménonville S., Orfila D., Béal M., Bertino B., Aubert J. (2018). A topical treatment containing heat-treated *Lactobacillus johnsonii* NCC 533 reduces *Staphylococcus aureus* adhesion and induces antimicrobial peptide expression in an in vitro reconstructed human epidermis model. Exp. Dermatol..

[141] Moro-García M.A., Alonso-Arias R., Baltadjieva M., Fernández Benítez C., Fernández Barrial M.A., Díaz Ruisánchez E. (2013). Oral supplementation with *Lactobacillus delbrueckii* subsp. bulgaricus 8481 enhances systemic immunity in elderly subjects. Age.

[142] Nocerino R., De Filippis F., Cecere G., Marino A., Micillo M., Di Scala C. (2020). The therapeutic efficacy of *Bifidobacterium animalis* subsp. lactis BB-12® in infant colic: a randomised, double blind, placebo-controlled trial, Aliment. Pharmacol. Ther..

[143] Schlee M., Harder J., Köten B., Stange E.F., Wehkamp J., Fellermann K. (2008). Probiotic Lactobacilli and VSL#3 induce enterocyte beta-defensin 2. Clin. Exp. Immunol..

[144] Hrdý J., Alard J., Couturier-Maillard A., Boulard O., Boutillier D., Delacre M. (2020). *Lactobacillus reuteri* 5454 and *Bifidobacterium animalis* ssp. lactis 5764 improve colitis while differentially impacting dendritic cells maturation and antimicrobial responses. Sci. Rep..

[145] Sánchez E., Nieto J.C., Vidal S., Santiago A., Martinez X., Sancho F.J. (2017). Fermented milk containing Lactobacillus paracasei subsp. paracasei CNCM I-1518 reduces bacterial translocation in rats treated with carbon tetrachloride. Sci. Rep..

[146] Aktas B., De Wolfe T.J., Safdar N., Darien B.J., Steele J.L. (2016). The Impact of *Lactobacillus casei* on the composition of the cecal microbiota and innate immune system is strain specific. PLoS One.

[147] Wang J., Zhang W., Wang S., Liu H., Zhang D., Wang Y. (2019). Swine-derived probiotic *Lactobacillus plantarum* modulates porcine intestinal endogenous host defense peptide synthesis through TLR2/MAPK/AP-1 signaling pathway. Front. Immunol..

[148] Wang J., Ji H., Wang S., Liu H., Zhang W., Zhang D. (2018). Probiotic *Lactobacillus plantarum* promotes intestinal barrier function by strengthening the epithelium and modulating gut microbiota. Front. Microbiol..

[149] Shen J., Zhang J., Zhao Y., Lin Z., Ji L., Ma X. (2022). Tibetan pig-derived probiotic *Lactobacillus amylovorus* SLZX20-1 improved intestinal function via producing enzymes and regulating intestinal microflora. Front. Nutr..

[150] Sudan S., Zhan X., Li J. (2022). A novel probiotic *Bacillus subtilis* strain confers cytoprotection to host pig intestinal epithelial cells during enterotoxic *Escherichia coli* infection. Microbiol. Spectr..

[151] Deng J., Li Y., Zhang J., Yang Q. (2013). Co-administration of *Bacillus subtilis* RJGP16 and *Lactobacillus salivarius* B1 strongly enhances the intestinal mucosal immunity of piglets. Res. Vet. Sci..

[152] Wang Y., Gong L., Wu Y.P., Cui Z.W., Wang Y.Q., Huang Y. (2019). Oral administration of *Lactobacillus rhamnosus* GG to newborn piglets augments gut barrier function in pre-weaning piglets. J. Zhejiang Univ. Sci. B..

[153] Terada T., Nii T., Isobe N., Yoshimura Y. (2020). Effects of probiotics *Lactobacillus reuteri* and *Clostridium butyricum* on the expression of toll-like receptors, pro- and anti-inflammatory cytokines, and antimicrobial peptides in broiler chick intestine. J. Poult. Sci..

[154] Souza R.F.S., Rault L., Seyffert N., Azevedo V., Le Loir Y., Even S. (2018). *Lactobacillus casei* BL23 modulates the innate immune response in *Staphylococcus aureus*-stimulated bovine mammary epithelial cells. Benef. Microbes..

[155] Bach A., Guasch I., Elcoso G., Chaucheyras-Durand F., Castex M., Fàbregas F. (2018). Changes in gene expression in the rumen and colon epithelia during the dry period through lactation of dairy cows and effects of live yeast supplementation. J. Dairy Sci..

[156] Chen K., Chen H., Faas M.M., de Haan B.J., Li J., Xiao P. (2017). Specific inulin-type fructan fibers protect against autoimmune diabetes by modulating gut immunity, barrier function, and microbiota homeostasis. Mol. Nutr. Food Res..

[157] Xie S.Z., Liu B., Ye H.Y., Li Q.M., Pan L.H., Zha X.Q. (2019). *Dendrobium huoshanense* polysaccharide regionally regulates intestinal mucosal barrier function and intestinal microbiota in mice. Carbohydr. Polym..

[158] Raqib R., Sarker P., Mily A., Alam N.H., Arifuzzaman A.S., Rekha R.S. (2012). Efficacy of sodium butyrate adjunct therapy in shigellosis: a randomized, double-blind, placebo-controlled clinical trial. BMC Infect. Dis..

[159] Hinnebusch B.F., Meng S., Wu J.T., Archer S.Y., Hodin R.A. (2002). The effects of short-chain fatty acids on human colon cancer cell phenotype are associated with histone hyperacetylation. J. Nutr..

[160] Zhao Y., Chen F., Wu W., Sun M., Bilotta A.J., Yao S. (2018). GPR43 mediates microbiota metabolite SCFA regulation of antimicrobial peptide expression in intestinal epithelial cells via activation of mTOR and STAT3. Mucosal Immunol.

[161] Chakraborty K., Ghosh S., Koley H., Mukhopadhyay A.K., Ramamurthy T., Saha D.R. (2008). Bacterial exotoxins downregulate cathelicidin (hCAP-18/LL-37) and human beta-defensin 1 (HBD-1) expression in the intestinal epithelial cells. Cell Microbiol.

[162] Selvaraj P., Prabhu Anand S., Harishankar M., Alagarasu K. (2009). Plasma 1,25 dihydroxy vitamin D3 level and expression of vitamin D receptor and cathelicidin in pulmonary tuberculosis. J. Clin. Immunol..

[163] Sato-Deguchi E., Imafuku S., Chou B., Ishii K., Hiromatsu K., Nakayama J. (2012). Topical vitamin D₃ analogues induce thymic stromal lymphopoietin and cathelicidin in psoriatic skin lesions. Br. J. Dermatol..

[164] Muehleisen B., Bikle D.D., Aguilera C., Burton D.W., Sen G.L., Deftos L.J., Gallo R.L. (2012). PTH/PTHrP and vitamin D control antimicrobial peptide expression and susceptibility to bacterial skin infection. Sci. Transl. Med..

[165] Kanda N., Hau C.S., Tada Y., Sato S., Watanabe S. (2012). Decreased serum LL-37 and vitamin D3 levels in atopic dermatitis: relationship between IL-31 and oncostatin M. Allergy.

[166] Finklea J.D., Grossmann R.E., Tangpricha V. (2011). Vitamin D and chronic lung disease: a review of molecular mechanisms and clinical studies. Adv. Nutr..

[167] Aibana O., Huang C.C., Aboud S., Arnedo-Pena A., Becerra M.C., Bellido-Blasco J.B. (2019). Vitamin D status and risk of incident tuberculosis disease: a nested case-control study, systematic review, and individual-participant data meta-analysis. PLoS Med.

[168] Ganmaa D., Uyanga B., Zhou X., Gantsetseg G., Delgerekh B., Enkhmaa D. (2020). Vitamin D supplements for prevention of tuberculosis infection and disease. N. Engl. J. Med..

[169] Tian G., Liang X., Chen D., Mao X., Yu J., Zheng P. (2016). Vitamin D3 supplementation alleviates Rotavirus infection in pigs and IPEC-J2 cells via regulating the autophagy signaling pathway. J. Steroid Biochem. Mol. Biol..

[170] Coyle C., Wheelhouse N., Jacques M., Longbottom D., Svoboda P., Pohl J. (2016). Ovine trophoblasts express cathelicidin host defence peptide in response to infection. J. Reprod. Immunol..

[171] Wei R., Dhawan P., Baiocchi R.A., Kim K.Y., Christakos S. (2019). PU.1 and epigenetic signals modulate 1,25-dihydroxyvitamin D3 and C/EBPalpha regulation of the human cathelicidin antimicrobial peptide gene in lung epithelial cells. J. Cell Physiol..

[172] Mielke L.A., Jones S.A., Raverdeau M., Higgs R., Stefanska A., Groom J.R. (2013). Retinoic acid expression associates with enhanced IL-22 production by γδ T cells and innate lymphoid cells and attenuation of intestinal inflammation. J. Exp. Med..

[173] Wu H., Zhang G., Minton J.E., Ross C.R., Blecha F. (2000). Regulation of cathelicidin gene expression: induction by lipopolysaccharide, interleukin-6, retinoic acid, and *Salmonella enterica* serovar typhimurium infection. Infect. Immun..

[174] Grubor B., Meyerholz D.K., Lazic T., DeMacedo M.M., Derscheid R.J., Hostetter J.M. (2006). Regulation of surfactant protein and defensin mRNA expression in cultured ovine type II pneumocytes by all-trans retinoic acid and VEGF. Int. J. Exp. Pathol..

[175] Ren M., Cai S., Zhou T., Zhang S., Li S., Jin E. (2019). Isoleucine attenuates infection induced by *E. coli* challenge through the modulation of intestinal endogenous antimicrobial peptide expression and the inhibition of the increase in plasma endotoxin and IL-6 in weaned pigs. Food Funct.

[176] Tsugami Y., Nii T., Isobe N. (2023). Valine treatment enhances antimicrobial component production in mammary epithelial cells and the milk of lactating goats without influencing the tight junction barrier. J. Mammary Gland Biol. Neoplasia.

[177] Tang Z., Shi B., Sun W., Yin Y., Chen Q., Mohamed T. (2020). Tryptophan promoted β-defensin-2 expression via the mTOR pathway and its metabolites: kynurenine banding to aryl hydrocarbon receptor in rat intestine. RSC Adv.

[178] Lan J., Dou X., Li J., Yang Y., Xue C., Wang C. (2020). L-Arginine ameliorates lipopolysaccharide-induced intestinal inflammation through inhibiting the TLR4/NF-κB and MAPK pathways and stimulating β-defensin expression in vivo and in vitro. J. Agric. Food Chem..

[179] Yin J., Yu F.S. (2010). LL-37 via EGFR transactivation to promote high glucose-attenuated epithelial wound healing in organ-cultured corneas. Invest. Ophthalmol. Vis. Sci..

[180] Lan C.C., Wu C.S., Huang S.M., Kuo H.Y., Wu I.H., Liang C.W. (2012). High-glucose environment reduces human beta-defensin-2 expression in human keratinocytes: implications for poor diabetic wound healing. Br. J. Dermatol..

[181] Wessels I., Fischer H.J., Rink L. (2021). Dietary and physiological effects of zinc on the immune system. Annu. Rev. Nutr..

[182] Kelly P., Feakins R., Domizio P., Murphy J., Bevins C., Wilson J. (2004). Paneth cell granule depletion in the human small intestine under infective and nutritional stress. Clin. Exp. Immunol..

[183] Zhong W., Wei X., Hao L., Lin T.D., Yue R., Sun X. (2020). Paneth cell dysfunction mediates alcohol-related steatohepatitis through promoting bacterial translocation in mice: role of zinc deficiency. Hepatology.

[184] Krisanaprakornkit S., Jotikasthira D., Dale B.A. (2003). Intracellular calcium in signaling human beta-defensin-2 expression in oral epithelial cells. J. Dent. Res..

[185] Mader J.S., Mookherjee N., Hancock R.E., Bleackley R.C. (2009). The human host defense peptide LL-37 induces apoptosis in a calpain- and apoptosis-inducing factor-dependent manner involving Bax activity. Mol. Cancer Res..

[186] Vylkova S., Nayyar N., Li W., Edgerton M. (2007). Human beta-defensins kill *Candida albicans* in an energy-dependent and salt-sensitive manner without causing membrane disruption. Antimicrob. Agents Chemother..

[187] Fuchs C.D., Trauner M. (2022). Role of bile acids and their receptors in gastrointestinal and hepatic pathophysiology. Nat. Rev. Gastroenterol. Hepatol..

[188] Wang S., Du Q., Meng X., Zhang Y. (2022). Natural polyphenols: a potential prevention and treatment strategy for metabolic syndrome. Food Funct.

[189] Tobin I., Zhang G. (2023). Regulation of host defense peptide synthesis by polyphenols. Antibiotics (Basel).

[190] Noguchi T., Shiba H., Komatsuzawa H., Mizuno N., Uchida Y., Ouhara K. (2003). Syntheses of prostaglandin E2 and E-cadherin and gene expression of beta-defensin-2 by human gingival epithelial cells in response to *Actinobacillus actinomycetemcomitans*. Inflammation.

[191] Perri F., Longo F., Giuliano M., Sabbatino F., Favia G., Ionna F. (2017). Epigenetic control of gene expression: potential implications for cancer treatment. Crit. Rev. Oncol. Hematol..

[192] Lyu W., Deng Z., Zhang G. (2023). High-throughput screening for epigenetic compounds that induce human beta-defensin 1 synthesis. Antibiotics. (Basel).

[193] Bouyahya A., El Hachlafi N., Aanniz T., Bourais I., Mechchate H., Benali T. (2022). Natural bioactive compounds targeting histone deacetylases in human cancers: recent updates. Molecules.

[194] Akone S.H., Ntie-Kang F., Stuhldreier F., Ewonkem M.B., Noah A.M., Mouelle S.E.M. (2020). Natural products impacting DNA methyltransferases and histone deacetylases. Front. Pharmacol..

[195] Barrier M.L., Myszor I.T., Sahariah P., Sigurdsson S., Carmena-Bargueño M., Pérez-Sánchez H. (2023). Aroylated phenylenediamine HO53 modulates innate immunity, histone acetylation and metabolism. Mol. Immunol..

[196] Gschwandtner M., Zhong S., Tschachler A., Mlitz V., Karner S., Elbe-Burger A. (2014). Fetal human keratinocytes produce large amounts of antimicrobial peptides: involvement of histone-methylation processes. J. Invest. Dermatol..

[197] Latif A., Shehzad A., Niazi S., Zahid A., Ashraf W., Iqbal M.W. (2023). Probiotics: mechanism of action, health benefits and their application in food industries. Front. Microbiol..

[198] Wehkamp J., Harder J., Wehkamp K., Wehkamp-von Meissner B., Schlee M., Enders C. (2004). NF-kappaB- and AP-1-mediated induction of human beta defensin-2 in intestinal epithelial cells by *Escherichia coli* Nissle 1917: a novel effect of a probiotic bacterium. Infect. Immun..

[199] Li X., Song L., Zhu S., Xiao Y., Huang Y., Hua Y. (2019). Two strains of *Lactobacilli* effectively decrease the colonization of VRE in a mouse model. Front. Cell Infect. Microbiol..

[200] Liu H., Hou C., Wang G., Jia H., Yu H., Zeng X. (2017). *Lactobacillus reuteri* I5007 modulates intestinal host defense peptide expression in the model of IPEC-J2 cells and neonatal piglets. Nutrients.

[201] Wang M., Wu H., Lu L., Jiang L., Yu Q. (2020). *Lactobacillus reuteri* promotes intestinal development and regulates mucosal immune function in newborn piglets. Front. Vet. Sci..

[202] Nii T., Jirapat J., Isobe N., Yoshimura Y. (2020). Effects of oral administration of *Lactobacillus reuteri* on mucosal barrier function in the digestive tract of broiler chicks. J. Poult. Sci..

[203] Jin X., Zhang M., Yang Y.F. (2019). *Saccharomyces cerevisiae* β-glucan-induced SBD-1 expression in ovine ruminal epithelial cells is mediated through the TLR-2-MyD88-NF-κB/MAPK pathway. Vet. Res. Commun..

[204] Strunk T., Hibbert J., Doherty D., Granland C., Trend S., Simmer K. (2017). Probiotics and antimicrobial protein and peptide levels in preterm infants, Acta. Paediatr..

[205] Wu S., Yoon S., Zhang Y.G., Lu R., Xia Y., Wan J. (2015). Vitamin D receptor pathway is required for probiotic protection in colitis. Am. J. Physiol. Gastrointest. Liver Physiol..

[206] Coussens A.K., Wilkinson R.J., Martineau A.R. (2015). Phenylbutyrate is bacteriostatic against *Mycobacterium tuberculosis* and regulates the macrophage response to infection, synergistically with 25-hydroxy-vitamin D3. PLoS. Pathog..

[207] van der Does A.M., Kenne E., Koppelaar E., Agerberth B., Lindbom L. (2014). Vitamin D_3_ and phenylbutyrate promote development of a human dendritic cell subset displaying enhanced antimicrobial properties. J. Leukoc. Biol..

[208] Yim S., Dhawan P., Ragunath C., Christakos S., Diamond G. (2007). Induction of cathelicidin in normal and CF bronchial epithelial cells by 1,25-dihydroxyvitamin D(3). J. Cyst. Fibros..

[209] Yang Q., Whitmore M.A., Robinson K., Lyu W., Zhang G. (2021). Butyrate, forskolin, and lactose synergistically enhance disease resistance by inducing the expression of the genes involved in innate host defense and barrier function. Antibiotics. (Basel)..

[210] Cederlund A., Nylén F., Miraglia E., Bergman P., Gudmundsson G.H., Agerberth B. (2014). Label-free quantitative mass spectrometry reveals novel pathways involved in LL-37 expression. J. Innate Immun.

